# Sodium Levels in Packaged Foods Sold in 14 Latin American and Caribbean Countries: A Food Label Analysis

**DOI:** 10.3390/nu11020369

**Published:** 2019-02-11

**Authors:** JoAnne Arcand, Adriana Blanco-Metzler, Karla Benavides Aguilar, Mary R. L’Abbe, Branka Legetic

**Affiliations:** 1 Faculty of Health Sciences, University of Ontario Institute of Technology 2000 Simcoe St N, Oshawa, ON L1H 7K4, Canada; joanne.arcand@uoit.ca; 2 Costa Rican Institute of Research and Training in Nutrition and Health (INCIENSA), Tres Rios Box 4-2250, Costa Rica; ablanco@inciensa.sa.cr (A.B.-M.); kbenavides@inciensa.sa.cr (K.B.A.); 3 Department of Nutritional Sciences, Faculty of Medicine, University of Toronto, Medical Sciences Building, 1 Kings College Circle, Rm 5368, Toronto, ON M5S 1A8, Canada; mary.labbe@utoronto.ca; 4 Independent Consultant, Washington, DC 20036, USA

**Keywords:** sodium, sodium reduction, sodium targets, food supply, food policy, public health, global health

## Abstract

Population-wide sodium reduction is a cost-effective approach to address the adverse health effects associated with excess sodium consumption. Latin American and Caribbean (LAC) countries consume excess dietary sodium. Packaged foods are a major contributor to sodium intake and a target for sodium reduction interventions. This study examined sodium levels in 12 categories of packaged foods sold in 14 LAC (*n* = 16,357). Mean sodium levels and percentiles were examined. Sodium levels were compared to regional sodium reduction targets. In this baseline analysis, 82% of foods met the regional target and 47% met the lower target. The greatest proportion of products meeting the regional target were uncooked pasta and noodles (98%), flavored cookies/crackers (97%), seasonings for sides/main dishes (96%), mayonnaise (94%), and cured/preserved meats (91%). A large proportion of foods met the lower target among uncooked pasta and noodles (88%), cooked pasta and noodles (88%), and meat/fish seasonings (88%). The highest the highest median sodium levels were among condiments (7778 mg/100 g), processed meats (870 mg/100 g), mayonnaise (755 mg/100 g), bread products (458 mg/100 g), cheese (643 mg/100 g), and snack foods (625 mg/100 g). These baseline data suggest that sodium reduction targets may need to be more stringent to enable effective lowering of sodium intake.

## 1. Introduction

Hypertension prevention and control in Latin American and Caribbean (LAC) countries is a priority action area for the World Health Organization (WHO) and Pan-American Health Organization (PAHO) to reduce the burden of cardiovascular disease and stroke. The current goal is to reduce hypertension prevalence to 35% by 2019 [1]. Hypertension is a major risk factor for cardiovascular disease (CVD) and accounts for nearly 1 in 5 deaths in LAC, with prevalence rates in many countries exceeding one-third of adults [2]. These rates are among the highest in the world.

Excess dietary sodium (salt) is a significant causal risk factor in the development of hypertension and is associated with cardiovascular and stroke morbidity and mortality [3,4,5]. To reduce the health burden associated with excess sodium intake, the WHO set a global target of reducing dietary salt intake by 30% by 2025 [6]. Worldwide, most developed countries consume excess sodium compared to the WHO’s recommended intake of 2000 mg sodium (5 g salt) per day [7]. Sodium intake in LAC is also high. For example, estimated sodium intake is 4407 mg (11.2 g salt)/day in Argentina [8,9], 4700 mg/day (12g salt/day) in Brazil [10], 4600 mg/day (11.5 g salt/day) in Costa Rica [11], and 7970 mg/day (19.9 g salt/day) in Colombia [12]. In Argentina, 65% to 70% of dietary sodium is derived from processed foods, with 25% from bread [13]. In Brazil, French bread (artisanal), soups, dairy and meat products are responsible for over 90% of sodium from processed foods [14], although more sodium comes from added salt and condiments. Over time, Costa Rica has seen a significant 15% increase in sodium intake, which is largely attributed to the higher intake of condiments and other processed foods [11]. In Costa Rica, income level was inversely related to sodium availability [11]. Data from Columbia show that 96 single food items contributed 72% of total dietary sodium in Colombians’ diet, with the most dietary sodium coming from bakery products (30.5%) [15]. Similar data and trends in sodium intake are found throughout the LAC region.

To facilitate the reduction and monitoring of sodium levels in foods produced and sold in LAC, regional sodium reduction targets were set for 12 categories (18 subcategories) of packaged foods that were commonly sold and consumed in LAC in 2015 [16]. These targets were adopted by the Salt Smart Consortium—a group of government, industry, and non-governmental organization stakeholders—in January 2015. The targets included a regional target (maximum) level and a lower target level, which were set at sodium reduction target levels similar to other jurisdictions. To date, there have been no studies examining sodium levels across LAC [16]. The purpose of this study was to conduct a cross-sectional examination of sodium levels in packaged foods sold in 14 LAC countries and determine the proportion of these foods that meet or exceed the 2015 established sodium reduction targets. This data will serve as critical baseline information that will be used for longitudinal monitoring of sodium levels in the food supply in LAC countries.

## 2. Materials and Methods

### 2.1. Study Design, Participants, and Data Collection

This cross-sectional survey was conducted between July 2015 and February 2016 in 14 participating countries: Argentina, Barbados, Brazil, Chile, Costa Rica, Cuba, Ecuador, Guatemala, Jamaica, Mexico, Paraguay, Panama, Peru, and Trinidad and Tobago.

Participating country research teams led by the presidents of the national branches of the Latin American Network of Food Composition Data Systems (LATINFOODS), who have expertise and experience in food composition, were trained to collect data on and develop a database of foods pertaining to the 12 food categories that comprise the regional sodium reduction targets. Data were collected according to a protocol that was established and approved by the PAHO/WHO Technical Advisory Group on Cardiovascular Diseases Prevention through Population-Wide Dietary Salt Reduction. Data on packaged foods was acquired by systematically capturing information on foods sold in grocery stores. The nutrition facts table data were recorded and photos of each food package were taken and stored. Grocery stores that had the greatest market share in each country were selected. Each country was required to collect a minimum number of samples for each food category: breads (*n* ≥100), soups (*n* ≥ 80), mayonnaise (*n* ≥ 30), cookies and biscuits (*n* ≥ 150), cakes (*n* ≥ 150), meat (i.e., sausages, cured meats, breaded meat) (*n* ≥ 200), breakfast cereals (*n* ≥ 100), dairy (*n* ≥ 150), butter and dairy spreads (*n* ≥ 50), snacks (*n* ≥ 200), pasta (*n* ≥ 50), and seasonings (*n* ≥ 100).

Data captured from each food packaged included the following: product name, number of servings per package, serving size, levels of nutrients on the nutrition facts table or equivalent (sodium, calories, sugar, etc.) per serving or per 100 g or both, as well as the presence of front of package information such as logos and claims. Where foods required nutrition information to be presented “as consumed,” the LATINFOODS database was used to create recipes (http://inta.cl/latinfoods/default). Specifically, recipes were required for the wet and dry soups and noodles in broth. This was a necessary step to ensure like-foods sold in different forms could be compared within the same food category (i.e., a soup fully prepared could be compared with a soup sold as a condensed product) when presented in a standardized format (mg/100 g). Once all data was entered, quality assurance measures were implemented. This included duplicate review and entry of food categories to ensure accurate classification, as well as ranking foods from highest to lowest sodium values (mg/serving and mg/100 g) to identify errors in typography, classification, or in recipes.

### 2.2. Analysis of Sodium Levels

Foods were classified into the food group categories established by the regional sodium reduction targets, which include major subcategories and minor subcategories [16]. The sodium content in foods was obtained from the nutrition facts table (mg/serving) and was converted to standardized units (mg/100 g). Means and standard deviations were calculated, as well as the 10th, 25th, 50th, and 75th percentiles and minimum and maximum levels. Standardized units (mg/100 g) were used to determine the proportion of products that met or exceeded the regional sodium target levels: the regional target level and the lower target level. Cheese was excluded from this analysis (*n* = 1911) since this food category does not have a sodium reduction target level. Continuous variables are presented as the mean ± standard deviation. Categorical variables are presented as frequency (percent).

## 3. Results

### 3.1. Mean Sodium Levels

This analysis included 16,357 foods across 12 major food categories and from 14 countries (Table S1). Overall, the highest median sodium levels per 100 g were among condiments (7778 mg/100 g, range: 0 to 51,670 mg/100 g), processed meats (870 mg/100 g, range: 389 to 7000 mg/100 g), mayonnaise (755 mg/100 g, range 470 to 4000 mg/100 g), bread products (458 mg/100 g, range: 190 to 4444 mg/100 g), cheese (643 mg/100 g, range: 274 to 14,740 mg/100 g), and snack foods (625 mg/100 g, range 211 to 42,860 mg/100 g) (Table 1). This data varied from country to country (Tables S2 and S3). Overall, there was substantive variability in sodium levels observed within certain food categories: condiments (0 to 51,670 mg/100 g), snack foods (0 to 42,860 mg/100 g), and cheeses (0 to 14,740 mg/100 g).

On examination of median levels across countries, high between-country variation was observed within food categories: condiments (434 mg/100 g in Cuba to 19,600 mg/100 g in Paraguay), pasta (2 mg/100 g in Cuba to 1651 mg/100 g in Brazil), butter (120 mg/100 g in Argentina to 786 mg/100 g in Peru), cheese (482 mg/100 g in Chile to 1146 mg/100 g in Ecuador), cookies and biscuits (183 mg/100 g in Chile to 677 mg/100 g in Peru), cakes (183 mg/100 g in Chile to 677 mg/100 g in Peru), mayonnaise (531 mg/100 g in Cuba to 975 mg/100 g in Brazil), and soups (94 mg/100 g in Cuba to 533 mg/100 g in Ecuador). However, a low to moderate level of between-country variation in sodium levels was observed for other food categories such as bread (410 mg/100 g in Barbados to 528 mg/100 g in Cuba), snack foods (458 mg/100 g in Chile to 764 mg/100 g in Brazil), processed meats (720 mg/100 g in Panama to 1035 mg/100 g in Brazil), and breakfast cereals (125 mg/100 g in Paraguay to 475 mg/100 g in Jamaica).

### 3.2. Proportion of Foods Meeting the Sodium Targets

Overall, across all countries and food categories, 82% of packaged foods met the regional target level and 47% met the lower target level. The greatest proportion of products meeting the regional target level was among uncooked noodles and pasta (98%), flavored cookies and crackers (97%), seasonings for sides and main dishes (96%), mayonnaise (94%), and cured and preserved meats (91%) (Table 2). The lowest proportion of foods meeting the regional targets were wet and dry soups (59%), bouillon cubes and powders (62%), cakes (64%), and breaded meat and poultry (65%). A few food categories had a large proportion of products meeting the lower target level: pasta and noodles, uncooked (88%), pasta and noodles, cooked (88%), and meat and fish seasonings (88%). However, several food categories had fewer products meeting the lower target: meats and sausages (25%), cakes (25%), breaded meat and poultry (28%), bread (34%), bouillon cubes and powders (37%), butter (37%), mayonnaise (38%), and snack foods (39%).

All countries had more than three-quarters of products meeting the target levels (Table 3). Two countries had >90% of food products meeting the regional targets, which included Chile (92%) and Cuba (90%). Countries with the lowest proportion of products meeting the regional targets were Brazil (77%), Costa Rica (77%), Guatemala (78%) and Trinidad and Tobago (79%). For five countries, more than half of food products contained sodium levels below the lower target: Cuba (59%), Peru (56%), Chile (55%), Paraguay (55%), and Ecuador (51%).

On examination of country-level data within individual food categories, some food categories had significant variation between countries in relation to the proportion of products that met the regional targets, while others did not (Table S4). For example, significant between-country variation was observed among breaded meat and poultry (range: 17% to 100%), noodles in broth (range: 25% to 100%), cakes (range: 31% to 100%), wet and dry soups (range: 18% to 81%), and bouillon cubes and powders (range: 33% to 93%). In contrast, a lower level of between-country variability was observed among pasta and noodles, uncooked (range: 94% to 100%), flavored cookies and crackers (range: 88% to 100%), mayonnaise (range: 86% to 100%), and breakfast cereals (range: 82% to 99%). Within-country variations were also observed in the proportion of foods meeting the sodium targets across food categories (Table S5).

## 4. Discussion

This is the first known comprehensive assessment of sodium levels in packaged foods sold in LAC countries. Although there was significant between-country variation in the mean sodium content of packaged foods across food categories, in 2015 an overwhelming proportion of packaged foods (83%) at baseline were already meeting the regional targets and approximately half of the foods met the lower target. Importantly, these are baseline data that will be used for longitudinal assessments of sodium in the food supply. These data may also be used to inform the revision of targets and timelines for sodium reduction in the region.

While sodium added through table salt or seasonings remains a significant source of dietary sodium in some LAC countries, the consumption of packaged and prepared foods is increasing [11,17]. This places significant importance on food supply interventions aimed at limiting or reducing the amount of sodium added to packaged and prepared foods; emphasizing the need for sodium reduction targets. The harmonized regional sodium targets were adopted by members of the PAHO-led Salt Smart Consortium in January 2015 [16]. The Consortium included a group of health, governmental, non-governmental, industry, and academic stakeholders from countries in the Americas. The intention of the harmonized targets was to provide a guideline for countries who do not yet have targets or timelines in place and to ensure regional food manufacturers have consistent reformulation goals, all for the benefit of reducing the population’s sodium intake. The harmonized targets were developed considering existing targets and timelines set in Argentina, Brazil, Canada, Chile, and the United Kingdom. The maximum level (mg/100 g), or upper limit, set by these countries was used to establish the regional target level. Where the regional target (maximum) level has been met, food manufacturers are encouraged to reformulate to the lower target level. The lower target level is an agreed upon level that is reflective of the average target levels in the reference countries; albeit, many of the regional lower target levels set slightly higher.

These data have similarities and differences compared to other baseline sodium assessments, which has important implications for the re-evaluation of sodium targets and timelines for the region. For example, in a baseline assessment of Canadian packaged foods, 25% of foods exceeded the maximum level [18,19], whereas 27% of foods in the current study exceeded the comparable regional target (maximum) level. However, baseline data in Canada showed that only 29% of foods met the sodium target goal level (Phase 3 level), while in the current analysis almost half (47%) of foods met the comparable lower target (goal) level. In this study, several key food categories, contributing a high proportion of dietary sodium (e.g., bread, meats and sausages, breaded meat and poultry), had fewer than one-third of products meet the lower target level. However, several food categories had more than half of food products already meeting the lower target including noodles in broth, cured and preserved meats, flavored cookies and crackers, breakfast cereals, pasta (cooked and uncooked), and meat/fish seasonings.

Given that the population’s sodium intake far exceeds recommendations in LAC, the data in this study point to the need for more stringent lower target levels to effectively achieve population-wide sodium reduction goals. In their report published in January 2015, the PAHO-led Salt Smart Consortium indicated a re-assessment of the targets every two years, in 2016 and 2018 and again in 2020. This is consistent with the approach taken by the United Kingdom [20]. To date, no known re-assessment of the targets has occurred. These data provide critical information that can be used in the evaluation and re-assessment of the sodium targets and timelines. In one published paper, the authors recommended that the maximum sodium target (i.e., regional level target) be based on the 70th percentile of products in a food category [21]. In Canada’s initial set of targets, which used sales-weighted averages for sodium in food categories, sodium reduction target levels were set at the 25th to 30th percentile while the maximum level was set at the 75th percentile [22].

At the time of data collection, there was regional between-country variance in regulatory approaches to reducing the sodium content of prepared and packaged food. Countries such as Brazil, Costa Rica, and Chile were working with the food industry to voluntarily reduce sodium levels in packaged foods. Countries such as Argentina and Paraguay have a mixed approach, with voluntary limits for many food categories and regulated limits on the sodium content of key foods such as bread products [23]. Longitudinal data on the sodium content of foods will allow for the analysis and comparisons of different regulatory approaches on changes to the sodium content of foods, on population sodium intake and on subsequent health outcomes. Such an analysis is needed since by 2017 more than 20 countries in the LAC region had national sodium reduction strategies with 12/20 countries having national level programs to address the sodium content of foods [24]. Additionally, many of these low- and middle-income countries have a high burden of hypertension and cardiovascular diseases; thus, a profound health and economic impact would be expected from implementing effective strategies that lower population sodium intake. 

It is important to discuss limitations of this study. Not every country collected the required sample size for each food category. However, data were systematically collected, and the smaller sample sizes reflect product availability in the market. Data in this study were also not sales-weighted. Sales-weighted averages would be considered ideal in assessing the relative contribution a product contributes to sodium levels based on market sales. However, such data is expensive, making it inaccessible, and is not available in all of the countries included in this study. The adopted harmonized targets also do not use sales-weighted averages; thus the analyses in this study are most appropriate. Finally, this analysis relied on nutrition information on the nutrition facts table. Regulations on the presence and variance of data on the nutrition facts table information vary across countries. In some countries, regulations permit up to 20% variance on values reported on the label, compared to the actual sodium content of foods. While the majority of foods have been found to comply with these regulatory requirements, a small proportion of foods may not [25]. This should be considered when interpreting the findings. Importantly, however, this data has been used in other research studies to monitor change over time and is currently the only data available to conduct surveillance activities.

## 5. Conclusions

In summary, these data provide a baseline assessment of sodium levels in packaged foods sold in LAC. A relatively high proportion of foods in the baseline analysis was already meeting the regional target levels, suggesting that targets in some food categories need to be made more stringent to enable effective lowering of population sodium intake. With longitudinal updating of this data, as part of a grant funding from the International Development Research Centre and PAHO, progress on sodium reformulation and subsequent reduction in population sodium intake and improved health outcomes can be tracked over time.

## Figures and Tables

**Table 1:** Overall levels of sodium in packaged foods by food category.

	*n*	Average Sodium mg/serving	Average Sodium mg/100 g	Sodium Percentiles (mg/100 g)					
				Min	10th	25th	50th	75th	Max
Wet and dry soups	1024	163 ± 160	402 ± 469	0	115	240	332	440	5900
Processed meats	2071	496 ± 414	928 ± 569	0	389	650	870	1091	7000
Bread products	1271	208 ± 148	465 ± 284	0	190	350	458	543	4444
Mayonnaise	337	111 ± 80	751 ± 295	0	470	571	755	893	4000
Cookies and biscuits	2169	113 ± 94	391 ± 296	0	111	199	315	500	3433
Cakes	1443	191 ± 129	383 ± 251	0	120	210	328	520	2743
Breakfast cereals	1457	110 ± 94	334 ± 280	0	11	113	327	486	3400
Cheese	1911	196 ± 172	739 ± 715	0	274	450	643	867	14,740
Butter	507	87 ± 151	592 ± 559	0	41	354	600	750	7636
Snack foods	2235	208 ± 303	724 ± 1041	0	211	400	625	905	42,860
Pasta	849	433 ± 500	493 ± 630	0	0	10	284	830	7000
Condiments	1083	684 ± 2113	10,791 ± 10,377	0	260	1176	7778	19,018	51,670

Data presented as mean ± standard deviation.

**Table 2:** Summary of the proportion of foods meeting the regional sodium reduction targets by food category.

	*n*	% Meeting Regional Target *n* (%)	% Exceeding Regional Target *n* (%)	% Meeting Lower Target *n* (%)
Overall	14,446	11,868 (82)	2578 (18)	6819 (47)
Wet and dry soups	817	485 (59)	332 (41)	353 (43)
Noodles in broth	207	169 (82)	38 (18)	114 (55)
Meats and sausages	1535	1329 (87)	206 (13)	378 (25)
Cured and preserved meats	320	290 (91)	30 (9)	213 (67)
Breaded meat and poultry	216	141 (65)	75 (35)	60 (28)
Bread products	1271	1053 (83)	218 (17)	434 (34)
Mayonnaise	337	317 (94)	20 (6)	128 (38)
Cookies and sweet cookies	1560	1406 (90)	154 (10)	750 (48)
Flavored cookies and crackers	609	591 (97)	18 (3)	362 (59)
Cakes	1443	919 (64)	524 (36)	356 (25)
Breakfast cereals	1457	1326 (91)	131 (9)	1114 (76)
Butter	507	428 (84)	79 (16)	186 (37)
Snacks	2235	1674 (75)	561 (25)	865 (39)
Pasta and noodles, uncooked	696	681 (98)	15 (2)	609 (88)
Pasta and noodles, as consumed	153	140 (92)	13 (8)	134 (88)
Seasonings for side and main dishes	390	375 (96)	15 (4)	286 (73)
Meat and fish seasonings	435	385 (89)	50 (11)	381 (86)
Bouillon cubes and powders	258	159 (62)	99 (38)	96 (37)

Data presented as *n* (%). Wet and dry soups, noodles and broth and bouillon cubes and powders are all presented “as consumed.”

**Table 3:** Summary of the proportion of products meeting or exceeding the regional sodium reduction targets by country.

	*n*	% Meeting Regional Target *n* (%)	% Exceeding Regional Target *n* (%)	% Meeting Lower Target *n* (%)
Overall	14,382	11,868 (82)	2578 (18)	6819 (47)
Argentina	1125	940 (84)	185 (16)	508 (45)
Barbados	1201	979 (82)	220 (18)	496 (41)
Brazil	1224	944 (77)	280 (23)	483 (39)
Chile	1178	1087 (92)	91 (8)	652 (55)
Costa Rica	1086	834 (77)	252 (23)	460 (43)
Cuba	209	188 (90)	21(10)	124 (59)
Ecuador	1177	976 (83)	201 (17)	604 (51)
Guatemala	944	734 (78)	207 (22)	430 (46)
Jamaica	907	731 (81)	176 (19)	399 (44)
Mexico	1267	1043 (82)	224 (18)	588 (46)
Panama	1339	1089 (81)	250 (19)	607 (45)
Paraguay	871	756 (87)	115 (13)	482 (55)
Peru	777	668 (86)	109 (14)	434 (56)
Trinidad and Tobago	1141	899 (79)	242 (21%)	542 (48)

Data presented as *n* (%).

## Supplementary Materials

The following are available online at http://www.mdpi.com/2072-6643/11/2/369/s1, Table S1: Number of packaged foods by food category and by country. Table S2: Detailed assessment of sodium levels in packaged foods by food category. Table S3: Detailed assessment sodium levels in packaged foods by country. Table S4: Detailed assessment of the proportion of foods meeting the regional sodium reduction targets by food category. Table S5: Detailed assessment of the proportion of foods meeting the regional sodium targets by country.
