# Supplementary material for: Sodium Levels in Packaged Foods Sold in 14 Latin American and Caribbean Countries: A Food Label Analysis
Source: Nutrients. 2019 Feb 11;11(2):369. doi: 10.3390/nu11020369 (PMC6412284; doi:10.3390/nu11020369)
Supplement: Supplementary file 1 [file nutrients-11-00369-s001.pdf]

Supplementary Materials

Sodium levels in packaged foods sold in 14 Latin American and Caribbean countries: A food label analysis

Table of Contents

Table S1. Number of packaged foods by Food Category and by Country 2

Table S2. Detailed assessment of sodium levels in packaged foods by Food Category 3

Table S3. Detailed assessment sodium levels in packaged foods by Country 11

Table S4. Detailed assessment of the proportion of foods meeting the Regional Sodium Reduction Targets by Food Category 19

Table S5. Detailed assessment of the proportion of foods meeting the Regional Sodium Targets by Country 30

Table S1. Number of packaged foods by Food Category and by Country

	Soup	Meat	Bread	Mayonnaise	Cookies and Biscuits	Cakes	Breakfast Cereals	Cheese	Butter	Snacks	Pasta	Condiments	Total
**Argentina**	79	212	123	18	165	127	88	150	23	123	96	71	**1275**
**Brazil**	80	200	101	33	150	154	100	158	50	198	51	107	**1382**
**Chile**	81	219	106	30	152	157	109	150	50	132	57	85	**1328**
**Costa Rica**	84	101	83	34	170	117	115	138	43	206	48	85	**1224**
**Cuba**	17	34	24	6	29	8	23	32	1	14	30	23	**241**
**Ecuador**	60	221	106	26	184	135	100	136	32	187	50	76	**1313**
**Guatemala**	79	60	68	29	147	96	100	164	68	181	66	50	**1108**
**Jamaica**	90	100	72	11	171	75	114	144	30	147	31	66	**1051**
**Mexico**	80	214	112	37	172	121	104	157	46	211	46	124	**1424**
**Panama**	71	187	137	25	199	129	190	170	51	207	64	79	**1509**
**Paraguay**	31	129	76	18	148	35	98	110	17	159	120	40	**981**
**Peru**	94	49	68	20	129	33	91	73	18	116	73	86	**850**
**Trinidad and Tobago**	98	179	95	20	193	127	112	177	28	152	60	77	**1318**
**Barbados**	80	166	100	30	160	129	113	152	50	202	57	114	**1353**
**Total**	**1024**	**2071**	**1271**	**337**	**2169**	**1443**	**1457**	**1911**	**507**	**2235**	**849**	**1083**	**16357**

Table S2. Detailed assessment of sodium levels in packaged foods by Food Category

	n	Average Sodium (mg/serving)	Average Sodium (mg/100g)	Sodium Percentiles (mg/100g)					
				Min	10th	25th	50th	75th	Max
**Soups**	**1024**	**163 ± 160**	**402 ± 469**	**0**	**115**	**240**	**332**	**440**	**5900**
Argentina	79	204 ± 341	884 ± 1417	2	25	247	327	964	5900
Brazil	80	110 ± 86	310 ± 203	1	71	119	309	421	794
Chile	81	101 ± 85	244 ± 125	35	71	154	205	366	439
Costa Rica	84	178 ± 144	417 ± 206	17	170	276	370	640	737
Cuba	17	35 ± 36	150 ± 139	15	30	86	94	124	460
Ecuador	60	184 ± 169	548 ± 260	3	274	387	533	760	1368
Guatemala	79	240 ± 195	422 ± 157	90	264	314	370	538	754
Jamaica	90	190 ± 122	420 ± 407	0	181	251	329	421	3467
Mexico	80	129 ± 111	268 ± 230	12	19	35	193	412	796
Panama	71	137 ± 112	341 ± 109	54	195	293	317	391	607
Paraguay	31	95 ± 91	333 ± 143	205	230	257	295	306	766
Peru	94	164 ± 110	339 ± 114	18	245	280	330	382	885
Trinidad and Tobago	98	203 ± 130	403 ± 176	43	189	281	372	527	771
Barbados	80	162 ± 128	355 ± 204	13	165	238	285	429	1053
**Meat**	**2071**	**496 ± 414**	**928 ± 569**	**0**	**389**	**650**	**870**	**1091**	**7000**
Argentina	212	468 ± 271	979 ± 559	30	457	700	850	1088	3500
Brazil	200	575 ± 240	1044 ± 388	274	617	812	1035	1226	3070
Chile	219	409 ± 255	995 ± 435	70	475	759	929	1144	2630
Costa Rica	101	605 ± 567	926 ± 611	31	406	511	827	1127	4200
Cuba	34	1086 ± 837	773 ± 478	95	116	428	780	908	1960
Ecuador	221	468 ± 288	992 ± 584	55	564	745	982	1120	6600
Guatemala	60	739 ± 1519	1067 ± 1003	0	1	700	940	1214	4500

	n	Average Sodium (mg/serving)	Average Sodium (mg/100g)	Sodium Percentiles (mg/100g)					
				Min	10th	25th	50th	75th	Max
Jamaica	100	499 ± 250	788 ± 310	47	400	632	810	982	1760
Mexico	214	627 ± 464	969 ± 528	114	450	691	900	1068	4545
Panama	187	453 ± 233	770 ± 688	12	140	420	720	905	5730
Paraguay	129	347 ± 214	793 ± 450	0	339	542	725	990	3000
Peru	49	315 ± 325	748 ± 1129	0	0	72	560	959	7000
Trinidad and Tobago	179	497 ± 443	868 ± 584	54	408	447	804	1035	4955
Barbados	166	428 ± 157	961 ± 429	0	560	713	855	1110	2320
**Bread**	**1271**	**208 ± 148**	**465 ± 284**	**0**	**190**	**350**	**458**	**543**	**4444**
Argentina	123	208 ± 146	452 ± 255	0	81	279	480	609	1200
Brazil	101	198 ± 51	417 ± 128	168	270	326	414	484	847
Chile	106	227 ± 89	428 ± 140	7	272	365	413	491	984
Costa Rica	83	232 ± 251	435 ± 314	0	0	215	473	591	1240
Cuba	24	312 ± 137	542 ± 76	381	473	479	528	601	696
Ecuador	106	192 ± 136	421 ± 200	0	123	329	427	520	1023
Guatemala	68	201 ± 159	519 ± 702	0	38	281	457	540	4444
Jamaica	72	257 ± 160	465 ± 137	0	298	415	481	516	969
Mexico	112	167 ± 122	603 ± 448	0	317	376	475	728	4000
Panama	137	178 ± 98	531 ± 221	10	320	440	500	610	1270
Paraguay	76	165 ± 78	398 ± 199	0	86	299	439	536	913
Peru	68	286 ± 281	503 ± 249	1	222	404	486	590	1300
Trinidad and Tobago	95	226 ± 138	421 ± 171	0	202	350	450	510	1000
Barbados	100	211 ± 106	403 ± 134	0	227	343	410	461	740
**Mayonnaise**	**337**	**111 ± 80**	**751 ± 295**	**0**	**470**	**570.67**	**754.55**	**892.86**	**4000**
Argentina	18	107 ± 26	856 ± 145	558	622	786	903	948	1067

	n	Average Sodium (mg/serving)	Average Sodium (mg/100g)	Sodium Percentiles (mg/100g)					
				Min	10th	25th	50th	75th	Max
Brazil	33	107 ± 24	881 ± 208	375	545	792	975	1025	1100
Chile	30	96 ± 25	759 ± 187	400	547	627	769	921	1031
Costa Rica	34	114 ± 26	754 ± 162	373	493	617	833	867	933
Cuba	6	80 ± 33	525 ± 140	354	374	413	531	633	696
Ecuador	26	106 ± 142	559 ± 206	11	393	438	539	643	1133
Guatemala	29	101 ± 37	702 ± 272	36	469	533	733	867	1250
Jamaica	11	84 ± 27	571 ± 165	333	393	393	633	706	767
Mexico	37	176 ± 182	763 ± 186	505	551	600	733	867	505
Panama	25	105 ± 25	745 ± 213	370	490	627	770	870	1400
Paraguay	18	109 ± 16	915 ± 120	610	741	883	942	988	1075
Peru	20	107 ± 58	789 ± 388	96	344	598	733	1000	2000
Trinidad and Tobago	20	88 ± 43	594 ± 298	0	303	400	647	713	1400
Barbados	30	105 ± 30	826 ± 626	500	518	600	670	800	4000
**Cookies and Biscuits**	**2169**	**113 ± 94**	**391 ± 296**	**0**	**111**	**199**	**315**	**500**	**3433**
Argentina	165	116 ± 113	377 ± 374	0	75	133	288	553	3433
Brazil	150	108 ± 72	356 ± 227	63	173	208	294	417	1443
Chile	152	80 ± 63	265 ± 198	6	95	130	228	331	1367
Costa Rica	170	122 ± 108	437 ± 343	0	139	215	318	560	1929
Cuba	29	42 ± 41	230 ± 173	16	43	43	197	306	700
Ecuador	184	87 ± 78	309 ± 256	0	33	120	250	437	1250
Guatemala	147	123 ± 129	457 ± 421	0	100	188	336	611	2400
Jamaica	171	142 ± 86	460 ± 241	86	197	299	400	606	1300
Mexico	172	100 ± 86	357 ± 288	0	57	165	283	448	1433
Panama	199	131 ± 86	479 ± 304	0	140	235	390	695	1640

	n	Average Sodium (mg/serving)	Average Sodium (mg/100g)	Sodium Percentiles (mg/100g)					
				Min	10th	25th	50th	75th	Max
Paraguay	148	88 ± 64	319 ± 211	0	134	199	266	374	1197
Peru	129	149 ± 99	432 ± 280	0	165	239	340	620	1643
Trinidad and Tobago	193	111 ± 109	393 ± 274	0	170	230	340	470	2270
Barbados	160	130 ± 82	451 ± 270	10	178	278	380	603	1670
**Cakes**	**1443**	**191 ± 129**	**383 ± 251**	**0**	**120**	**210**	**328**	**520**	**2743**
Argentina	127	146 ± 91	323 ± 204	3	81	214	260	435	820
Brazil	154	133 ± 53	324 ± 153	55	144	200	315	443	860
Chile	157	149 ± 110	231 ± 180	21	73	113	183	313	1460
Costa Rica	117	180 ± 114	385 ± 244	22	132	195	297	572	971
Cuba	8	65 ± 31	225 ± 84	75	132	198	214	303	320
Ecuador	135	192 ± 230	331 ± 333	0	50	148	258	430	2743
Guatemala	96	212 ± 115	470 ± 273	22	165	253	402	701	1483
Jamaica	75	302 ± 93	540 ± 224	125	267	330	595	721	947
Mexico	121	171 ± 132	369 ± 232	0	143	238	324	438	1371
Panama	129	222 ± 121	405 ± 235	0	124	270	380	530	1130
Paraguay	35	198 ± 92	472 ± 176	120	200	368	496	577	765
Peru	33	172 ± 134	425 ± 301	0	104	200	677	104	1465
Trinidad and Tobago	127	240 ± 102	433 ± 255	80	190	260	330	660	1580
Barbados	129	235 ± 96	505 ± 258	40	200	300	420	720	1250
**Breakfast Cereals**	**1457**	**110 ± 94**	**334 ± 280**	**0**	**11**	**113**	**327**	**486**	**3400**
Argentina	88	95 ± 76	318 ± 253	0	46	113	277	518	1100
Brazil	100	73 ± 66	224 ± 217	0	18	76	166	327	1500
Chile	109	69 ± 57	213 ± 175	1	7	50	188	324	642

	n	Average Sodium (mg/serving)	Average Sodium (mg/100g)	Sodium Percentiles (mg/100g)					
				Min	10th	25th	50th	75th	Max
Costa Rica	115	140 ± 63	433 ± 185	44	240	315	400	550	1067
Cuba	23	175 ± 178	602 ± 708	0	150	297	450	568	2760
Ecuador	100	84 ± 149	283 ± 498	0	0	19	200	384	3400
Guatemala	100	133 ± 56	412 ± 188	0	183	300	433	533	800
Jamaica	114	168 ± 93	456 ± 223	0	141	335	457	575	1179
Mexico	104	103 ± 65	323 ± 204	0	27	99	356	467	1073
Panama	190	118 ± 69	344 ± 201	0	10	220	355	493	810
Paraguay	98	60 ± 63	201 ± 210	0	12	69	125	269	977
Peru	91	72 ± 91	251 ± 276	0	2	7	229	378	1395
Trinidad and Tobago	112	147 ± 130	406 ± 245	0	85	265	388	534	1280
Barbados	113	122 ± 97	356 ± 316	0	10	70	370	510	1680
**Cheese**	**1911**	**196 ± 172**	**739 ± 715**	**0**	**274**	**450**	**643**	**867**	**14740**
Argentina	150	194 ± 108	679 ± 731	27	236	467	583	803	8800
Brazil	158	200 ± 116	664 ± 400	50	327	400	562	830	3000
Chile	150	144 ± 82	603 ± 396	42	261	340	482	795	2230
Costa Rica	138	282 ± 412	842 ± 845	0	300	500	627	960	6800
Cuba	32	162 ± 14	838 ± 439	39	339	405	900	1122	1930
Ecuador	136	178 ± 100	627 ± 349	17	208	417	567	808	1900
Guatemala	164	225 ± 236	1089 ± 1840	20	312	530	670	1089	14740
Jamaica	144	181 ± 106	738 ± 385	36	289	536	643	1012	2000
Mexico	157	210 ± 168	608 ± 325	35	247	400	600	718	1789
Panama	170	217 ± 163	752 ± 415	40	266	560	680	890	2360
Paraguay	110	209 ± 108	718 ± 347	0	373	503	687	833	1900
Peru	73	163 ± 117	686 ± 535	3	36	360	679	900	3038
Trinidad and Tobago	177	168 ± 90	772 ± 388	0	302	600	1146	964	2000

	n	Average Sodium (mg/serving)	Average Sodium (mg/100g)	Sodium Percentiles (mg/100g)					
				Min	10th	25th	50th	75th	Max
Barbados	152	177 ± 84	743 ± 335	110	321	600	680	900	1640
**Butter**	**507**	**87 ± 151**	**592 ± 559**	**0**	**41**	**354**	**600**	**750**	**7636**
Argentina	23	21 ± 23	199 ± 228	0	20	45	120	230	720
Brazil	50	71 ± 27	710 ± 267	170	396	563	705	853	1750
Chile	50	39 ± 18	545 ± 221	9	280	415	571	617	1171
Costa Rica	43	125 ± 302	553 ± 362	0	86	368	600	679	2000
Cuba	1	36 ± 0	511 ± 0	511	511	511	511	511	511
Ecuador	32	103 ± 186	732 ± 1330	0	0	27	500	946	7636
Guatemala	68	84 ± 66	672 ± 681	0	14	428	705	785	4200
Jamaica	30	79 ± 42	595 ± 251	0	348	525	625	714	1000
Mexico	46	30 ± 72	297 ± 259	0	10	159	200	495	1020
Panama	51	84 ± 37	594 ± 258	0	0	595	640	750	930
Paraguay	17	48 ± 34	484 ± 339	96	132	200	470	670	1110
Peru	18	141 ± 179	1079 ± 1238	20	120	413	786	1173	5000
Trinidad and Tobago	28	281 ± 366	781 ± 400	0	459	573	714	841	1733
Barbados	50	82 ± 53	564 ± 286	0	200	473	620	710	1790
**Snacks**	**2235**	**208 ± 303**	**724 ± 1041**	**0**	**211**	**400**	**625**	**905**	**42860**
Argentina	123	223 ± 272	863 ± 1043	0	174	423	608	900	8000
Brazil	198	196 ± 122	812 ± 484	20	347	517	764	1010	3516
Chile	132	117 ± 71	443 ± 271	0	12	267	458	623	1300
Costa Rica	206	195 ± 157	765 ± 579	0	216	395	643	956	5013
Cuba	14	229 ± 83	622 ± 179	330	365	544	675	699	1023
Ecuador	187	185 ± 162	591 ± 483	0	117	271	467	786	3214
Guatemala	181	282 ± 880	774 ± 701	0	178	410	689	964	7000

	n	Average Sodium (mg/serving)	Average Sodium (mg/100g)	Sodium Percentiles (mg/100g)					
				Min	10th	25th	50th	75th	Max
Jamaica	147	210 ± 144	671 ± 436	11	217	384	607	913	3393
Mexico	211	269 ± 267	812 ± 518	0	324	500	714	975	3166
Panama	207	214 ± 151	735 ± 464	0	262	410	670	1000	3230
Paraguay	159	184 ± 113	670 ± 425	0	34	400	610	944	2333
Peru	116	162 ± 117	584 ± 399	0	182	377	537	700	3000
Trinidad and Tobago	152	213 ± 135	676 ± 357	0	250	428	635	933	1710
Barbados	202	211 ± 228	907 ± 2997	0	252	430	635	910	42860
**Pasta**	**849**	**433 ± 500**	**493 ± 630**	**0**	**0**	**10**	**284**	**830**	**7000**
Argentina	96	264 ± 360	296 ± 412	0	6	10	14	682	1572
Brazil	51	1309 ± 262	1531 ± 344	244	1070	1363	1651	1765	1942
Chile	57	155 ± 309	236 ± 474	2	2	2	10	25	1742
Costa Rica	48	597 ± 596	840 ± 640	0	0	469	810	1045	2800
Cuba	30	4 ± 3	4 ± 4	0	2	2	2	5	20
Ecuador	50	326 ± 610	403 ± 658	0	2	14	265	532	3295
Guatemala	66	132 ± 297	169 ± 354	0	0	0	12	125	1400
Jamaica	31	608 ± 219	724 ± 382	234	300	308	777	950	1861
Mexico	46	696 ± 346	669 ± 401	25	273	332	505	929	1500
Panama	64	533 ± 734	357 ± 694	0	0	0	220	350	3700
Paraguay	120	497 ± 422	545 ± 513	0	513	513	513	513	2035
Peru	73	96 ± 465	218 ± 843	0	0	0	5	129	7000
Trinidad and Tobago	60	279 ± 320	250 ± 407	0	0	0	10	323	2140
Barbados	57	656 ± 182	954 ± 431	0	346	830	1000	1070	2230
**Condiments**	**1083**	**716 ± 2230**	**11271 ± 10448**	**0**	**220**	**1300**	**8889**	**19576**	**40000**

	n	Average Sodium (mg/serving)	Average Sodium (mg/100g)	Sodium Percentiles (mg/100g)					
				Min	10th	25th	50th	75th	Max
Argentina	71	555 ± 372	12977 ± 9391	0	900	2670	13410	19831	33810
Brazil	107	961 ± 403	20184 ± 8218	1852	8616	15797	18700	26256	38000
Chile	85	389 ± 320	9220 ± 8041	9	577	2317	8602	12313	33100
Costa Rica	85	512 ± 415	12213 ± 9936	0	7	3389	10000	19800	33000
Cuba	23	521 ± 580	4528 ± 7502	0	123	227	434	6620	24000
Ecuador	76	247 ± 305	5542 ± 8397	0	0	188	1460	6060	39300
Guatemala	50	459 ± 277	6179 ± 7525	0	400	818	2200	9200	25500
Jamaica	66	242 ± 158	12068 ± 9245	0	2188	5314	8889	17725	36000
Mexico	124	1229 ± 2911	13645 ± 9553	0	730	5675	13803	20632	38000
Panama	79	1029 ± 3450	3630 ± 5187	0	416	645	920	5415	25400
Paraguay	40	842 ± 591	20509 ± 9173	5600	8976	15350	19600	20995	38000
Peru	86	456 ± 637	9302 ± 10747	0	20	766	3311	20288	32670
Trinidad and Tobago	77	1256 ± 5795	9895 ± 13647	0	16	31	2600	14286	40000
Barbados	114	417 ± 325	6710 ± 8785	0	493	933	2945	8668	51670

Data presented as mean ± standard deviation.

Table S3. Detailed assessment sodium levels in packaged foods by Country

	n	Average Sodium (mg/serving)	Average Sodium (mg/100g)	Sodium Percentiles (mg/100g)					
				Min	10th	25th	50th	75th	Max
**Argentina**	**1275**								
Soup	79	204 ± 341	884 ± 1417	2	25	247	327	964	5900
Meat	212	468 ± 271	979 ± 559	30	457	700	850	1088	3500
Bread	123	208 ± 146	452 ± 255	0	81	279	480	609	1200
Mayonnaise	18	107 ± 26	856 ± 145	558	622	786	903	948	1067
Cookies and Biscuits	165	116 ± 113	377 ± 374	0	75	133	288	553	3433
Cakes	127	146 ± 91	323 ± 204	3	81	214	260	435	820
Breakfast Cereals	88	95 ± 76	318 ± 253	0	46	113	277	518	1100
Cheese	150	194 ± 108	679 ± 731	27	236	467	583	803	8800
Butter	23	21 ± 23	199 ± 228	0	20	45	120	230	720
Snacks	123	223 ± 272	863 ± 1043	0	174	423	608	900	8000
Pasta	96	264 ± 360	296 ± 412	0	6	10	14	682	1572
Condiments	71	555 ± 372	12977 ± 9391	0	900	2670	13410	19831	33810
**Brazil**	**1382**								
Soup	80	110 ± 86	310 ± 203	1	71	119	309	421	794
Meat	200	575 ± 240	1044 ± 388	274	617	812	1035	1226	3070
Bread	101	198 ± 51	417 ± 128	168	270	326	414	484	847
Mayonnaise	33	107 ± 24	881 ± 208	375	545	792	975	1025	1100
Cookies and Biscuits	150	108 ± 72	356 ± 227	63	173	208	294	417	1443
Cakes	154	133 ± 53	324 ± 153	55	144	200	315	443	860
Breakfast Cereals	100	73 ± 66	224 ± 217	0	18	76	166	327	1500

	n	Average Sodium (mg/serving)	Average Sodium	Sodium Percentiles (mg/100g)					
			(mg/100g)	Min	10th	25th	50th	75th	Max
Cheese	158	200 ± 116	664 ± 400	50	327	400	562	830	3000
Butter	50	71 ± 27	710 ± 267	170	396	563	705	853	1750
Snacks	198	196 ± 122	812 ± 484	20	347	517	764	1010	3516
Pasta	51	1309 ± 262	1531 ± 344	244	1070	1363	1651	1765	1942
Condiments	107	961 ± 403	20184 ± 8218	1852	8616	15797	18700	26256	38000
**Chile**	**1328**								
Soup	81	101 ± 85	244 ± 125	35	71	154	205	366	439
Meat	219	409 ± 255	995 ± 435	70	475	759	929	1144	2630
Bread	106	227 ± 89	428 ± 140	7	272	365	413	491	984
Mayonnaise	30	96 ± 25	759 ± 187	400	547	627	769	921	1031
Cookies and Biscuits	152	80 ± 63	265 ± 198	6	95	130	228	331	1367
Cakes	157	149 ± 110	231 ± 180	21	73	113	183	313	1460
Breakfast Cereals	109	69 ± 57	213 ± 175	1	7	50	188	324	642
Cheese	150	144 ± 82	603 ± 396	42	261	340	482	795	2230
Butter	50	39 ± 18	545 ± 221	9	280	415	571	617	1171
Snacks	132	117 ± 71	443 ± 271	0	12	267	458	623	1300
Pasta	57	155 ± 309	236 ± 474	2	2	2	10	25	1742
Condiments	85	389 ± 320	9220 ± 8041	9	577	2317	8602	12313	33100
**Costa Rica**	**1224**								
Soup	84	178 ± 144	417 ± 206	17	170	276	370	640	737
Meat	101	605 ± 567	926 ± 611	31	406	511	827	1127	4200
Bread	83	232 ± 251	435 ± 314	0	0	215	473	591	1240
Mayonnaise	34	114 ± 26	754 ± 162	373	493	617	833	867	933
Cookies and Biscuits	170	122 ± 108	437 ± 343	0	139	215	318	560	1929

	n	Average Sodium (mg/serving)	Average Sodium (mg/100g)	Sodium Percentiles (mg/100g)					
				Min	10th	25th	50th	75th	Max
Cakes	117	180 ± 114	385 ± 244	22	132	195	297	572	971
Breakfast Cereals	115	140 ± 63	433 ± 185	44	240	315	400	550	1067
Cheese	138	282 ± 412	842 ± 845	0	300	500	627	960	6800
Butter	43	125 ± 302	553 ± 362	0	86	368	600	679	2000
Snacks	206	195 ± 157	765 ± 579	0	216	395	643	956	5013
Pasta	48	597 ± 596	840 ± 640	0	0	469	810	1045	2800
Condiments	85	512 ± 415	12213 ± 9936	0	7	3389	10000	19800	33000
**Cuba**	**241**								
Soup	17	35 ± 36	150 ± 139	15	30	86	94	124	460
Meat	34	1086 ± 837	773 ± 478	95	116	428	780	908	1960
Bread	24	312 ± 137	542 ± 76	381	473	479	528	601	696
Mayonnaise	6	80 ± 33	525 ± 140	354	374	413	531	633	696
Cookies and Biscuits	29	42 ± 41	230 ± 173	16	43	43	197	306	700
Cakes	8	65 ± 31	225 ± 84	75	132	198	214	303	320
Breakfast Cereals	23	175 ± 178	602 ± 708	0	150	297	450	568	2760
Cheese	32	162 ± 14	838 ± 439	39	339	405	900	1122	1930
Butter	1	36 ± 0	511 ± 0	511	511	511	511	511	511
Snacks	14	229 ± 83	622 ± 179	330	365	544	675	699	1023
Pasta	30	4 ± 3	4 ± 4	0	2	2	2	5	20
Condiments	23	521 ± 580	5709 ± 8323	0	140	249	511	10015	24000
**Ecuador**	**1313**								
Soup	60	184 ± 169	548 ± 260	3	274	387	533	760	1368
Meat	221	468 ± 288	992 ± 584	55	564	745	982	1120	6600
Bread	106	192 ± 136	421 ± 200	0	123	329	427	520	1023

	n	Average Sodium (mg/serving)	Average Sodium	Sodium Percentiles (mg/100g)					
			(mg/100g)	Min	10th	25th	50th	75th	Max
Mayonnaise	26	106 ± 142	559 ± 206	11	393	438	539	643	1133
Cookies and Biscuits	184	87 ± 78	309 ± 256	0	33	120	250	437	1250
Cakes	135	192 ± 230	331 ± 333	0	50	148	258	430	2743
Breakfast Cereals	100	84 ± 149	283 ± 498	0	0	19	200	384	3400
Cheese	136	178 ± 100	627 ± 349	17	208	417	567	808	1900
Butter	32	103 ± 186	732 ± 1330	0	0	27	500	946	7636
Snacks	187	185 ± 162	591 ± 483	0	117	271	467	786	3214
Pasta	50	326 ± 610	403 ± 658	0	2	14	265	532	3295
Condiments	76	247 ± 305	5542 ± 8397	0	0	188	1460	6060	39300
**Guatemala**	**1108**								
Soup	79	240 ± 195	422 ± 157	90	264	314	370	538	754
Meat	60	739 ± 1519	1067 ± 1003	0	1	700	940	1214	4500
Bread	68	201 ± 159	519 ± 702	0	38	281	457	540	4444
Mayonnaise	29	101 ± 37	702 ± 272	36	469	533	733	867	1250
Cookies and Biscuits	147	123 ± 129	457 ± 421	0	100	188	336	611	2400
Cakes	96	212 ± 115	470 ± 273	22	165	253	402	701	1483
Breakfast Cereals	100	133 ± 56	412 ± 188	0	183	300	433	533	800
Cheese	164	225 ± 236	1089 ± 1840	20	312	530	670	1089	14740
Butter	68	84 ± 66	672 ± 681	0	14	428	705	785	4200
Snacks	181	282 ± 880	774 ± 701	0	178	410	689	964	7000
Pasta	66	132 ± 297	169 ± 354	0	0	0	12	125	1400
Condiments	50	459 ± 277	6179 ± 7525	0	400	818	2200	9200	25500
**Jamaica**	**1051**								
Soup	90	190 ± 122	420 ± 407	0	181	251	329	421	3467

	n	Average Sodium (mg/serving)	Average Sodium (mg/100g)	Sodium Percentiles (mg/100g)					
				Min	10th	25th	50th	75th	Max
Meat	100	499 ± 250	788 ± 310	47	400	632	810	982	1760
Bread	72	257 ± 160	465 ± 137	0	298	415	481	516	969
Mayonnaise	11	84 ± 27	571 ± 165	333	393	393	633	706	767
Cookies and Biscuits	171	142 ± 86	460 ± 241	86	197	299	400	606	1300
Cakes	75	302 ± 93	540 ± 224	125	267	330	595	721	947
Breakfast Cereals	114	168 ± 93	456 ± 223	0	141	335	457	575	1179
Cheese	144	181 ± 106	738 ± 385	36	289	536	643	1012	2000
Butter	30	79 ± 42	595 ± 251	0	348	525	625	714	1000
Snacks	147	210 ± 144	671 ± 436	11	217	384	607	913	3393
Pasta	31	608 ± 219	724 ± 382	234	300	308	777	950	1861
Condiments	66	242 ± 158	12068 ± 9245	0	2188	5314	8889	17725	36000
**Mexico**	**1424**								
Soup	80	129 ± 111	268 ± 230	12	19	35	193	412	796
Meat	214	627 ± 464	969 ± 528	114	450	691	900	1068	4545
Bread	112	167 ± 122	603 ± 448	0	317	376	475	728	4000
Mayonnaise	37	176 ± 182	763 ± 186	505	551	600	733	867	505
Cookies and Biscuits	172	100 ± 86	357 ± 288	0	57	165	283	448	1433
Cakes	121	171 ± 132	369 ± 232	0	143	238	324	438	1371
Breakfast Cereals	104	103 ± 65	323 ± 204	0	27	99	356	467	1073
Cheese	157	210 ± 168	608 ± 325	35	247	400	600	718	1789
Butter	46	30 ± 72	297 ± 259	0	10	159	200	495	1020
Snacks	211	269 ± 267	812 ± 518	0	324	500	714	975	3166
Pasta	46	696 ± 346	669 ± 401	25	273	332	505	929	1500
Condiments	124	1229 ± 2911	13645 ± 9553	0	730	5675	13803	20632	38000

	n	Average Sodium (mg/serving)	Average Sodium (mg/100g)	Sodium Percentiles (mg/100g)					
				Min	10th	25th	50th	75th	Max
**Panama**	**1509**								
Soup	71	137 ± 112	341 ± 109	54	195	293	317	391	607
Meat	187	453 ± 233	770 ± 688	12	140	420	720	905	5730
Bread	137	178 ± 98	531 ± 221	10	320	440	500	610	1270
Mayonnaise	25	105 ± 25	745 ± 213	370	490	627	770	870	1400
Cookies and Biscuits	199	131 ± 86	479 ± 304	0	140	235	390	695	1640
Cakes	129	222 ± 121	405 ± 235	0	124	270	380	530	1130
Breakfast Cereals	190	118 ± 69	344 ± 201	0	10	220	355	493	810
Cheese	170	217 ± 163	752 ± 415	40	266	560	680	890	2360
Butter	51	84 ± 37	594 ± 258	0	0	595	640	750	930
Snacks	207	214 ± 151	735 ± 464	0	262	410	670	1000	3230
Pasta	64	533 ± 734	357 ± 694	0	0	0	220	350	3700
Condiments	79	1029 ± 3450	3630 ± 5187	0	416	645	920	5415	25400
**Paraguay**	**981**								
Soup	31	95 ± 91	333 ± 143	205	230	257	295	306	766
Meat	129	347 ± 214	793 ± 450	0	339	542	725	990	3000
Bread	76	165 ± 78	398 ± 199	0	86	299	439	536	913
Mayonnaise	18	109 ± 16	915 ± 120	610	741	883	942	988	1075
Cookies and Biscuits	148	88 ± 64	319 ± 211	0	134	199	266	374	1197
Cakes	35	198 ± 92	472 ± 176	120	200	368	496	577	765
Breakfast Cereals	98	60 ± 63	201 ± 210	0	12	69	125	269	977
Cheese	110	209 ± 108	718 ± 347	0	373	503	687	833	1900
Butter	17	48 ± 34	484 ± 339	96	132	200	470	670	1110
Snacks	159	184 ± 113	670 ± 425	0	34	400	610	944	2333

	n	Average Sodium (mg/serving)	Average Sodium (mg/100g)	Sodium Percentiles (mg/100g)					
				Min	10th	25th	50th	75th	Max
Pasta	120	497 ± 422	545 ± 513	0	513	513	513	513	2035
Condiments	40	842 ± 591	20509 ± 9173	5600	8976	15350	19600	20995	38000
**Peru**	**850**								
Soup	94	164 ± 110	339 ± 114	18	245	280	330	382	885
Meat	49	315 ± 325	748 ± 1129	0	0	72	560	959	7000
Bread	68	286 ± 281	503 ± 249	1	222	404	486	590	1300
Mayonnaise	20	107 ± 58	789 ± 388	96	344	598	733	1000	2000
Cookies and Biscuits	129	149 ± 99	432 ± 280	0	165	239	340	620	1643
Cakes	33	172 ± 134	425 ± 301	0	104	200	677	104	1465
Breakfast Cereals	91	72 ± 91	251 ± 276	0	2	7	229	378	1395
Cheese	73	163 ± 117	686 ± 535	3	36	360	679	900	3038
Butter	18	141 ± 179	1079 ± 1238	20	120	413	786	1173	5000
Snacks	116	162 ± 117	584 ± 399	0	182	377	537	700	3000
Pasta	73	96 ± 465	218 ± 843	0	0	0	5	129	7000
Condiments	86	456 ± 637	9302 ± 10747	0	20	766	3311	20288	32670
**Trinidad and Tobago**	**1318**								
Soup	98	203 ± 130	403 ± 176	43	189	281	372	527	771
Meat	179	497 ± 443	868 ± 584	54	408	447	804	1035	4955
Bread	95	226 ± 138	421 ± 171	0	202	350	450	510	1000
Mayonnaise	20	88 ± 43	594 ± 298	0	303	400	647	713	1400
Cookies and Biscuits	193	111 ± 109	393 ± 274	0	170	230	340	470	2270
Cakes	127	240 ± 102	433 ± 255	80	190	260	330	660	1580

	n	Average Sodium (mg/serving)	Average Sodium	Sodium Percentiles (mg/100g)					
			(mg/100g)	Min	10th	25th	50th	75th	Max
Breakfast Cereals	112	147 ± 130	406 ± 245	0	85	265	388	534	1280
Cheese	177	168 ± 90	772 ± 388	0	302	600	1146	964	2000
Butter	28	281 ± 366	781 ± 400	0	459	573	714	841	1733
Snacks	152	213 ± 135	676 ± 357	0	250	428	635	933	1710
Pasta	60	279 ± 320	250 ± 407	0	0	0	10	323	2140
Condiments	77	1256 ± 5795	9895 ± 13647	0	16	31	2600	14286	40000
**Barbados**	**1353**								
Soup	80	162 ± 128	355 ± 204	13	165	238	285	429	1053
Meat	166	428 ± 157	961 ± 429	0	560	713	855	1110	2320
Bread	100	211 ± 106	403 ± 134	0	227	343	410	461	740
Mayonnaise	30	105 ± 30	826 ± 626	500	518	600	670	800	4000
Cookies and Biscuits	160	130 ± 82	451 ± 270	10	178	278	380	603	1670
Cakes	129	235 ± 96	505 ± 258	40	200	300	420	720	1250
Breakfast Cereals	113	122 ± 97	356 ± 316	0	10	70	370	510	1680
Cheese	152	177 ± 84	743 ± 335	110	321	600	680	900	1640
Butter	50	82 ± 53	564 ± 286	0	200	473	620	710	1790
Snacks	202	211 ± 228	907 ± 2997	0	252	430	635	910	42860
Pasta	57	656 ± 182	954 ± 431	0	346	830	1000	1070	2230
Condiments	114	417 ± 325	6710 ± 8785	0	493	933	2945	8668	51670

Data presented as mean ± standard deviation.

Table S4. Detailed assessment of the proportion of foods meeting the Regional Sodium Reduction Targets by Food Category

	n	Regional Target (mg Na/100 g)	% Meeting Regional Target	% Exceeding Regional Target	Lower Target (mg Na/100 g)	% Meeting Lower Target
**Wet and Dry Soups**	**817**		**485 (59%)**	**332 (41%)**		**353 (43%)**
Argentina	79	**360**	51 (65%)	28 (35%)	**306**	30 (38%)
Brazil	73	**360**	49 (67%)	24 (33%)	**306**	38 (52%)
Chile	69	**360**	47 (68%)	22 (32%)	**306**	44 (64%)
Costa Rica	71	**360**	34 (48%)	37 (52%)	**306**	23 (32%)
Cuba	16	**360**	13 (81%)	3 (19%)	**306**	12 (75%)
Ecuador	56	**360**	10 (18%)	46 (82%)	**306**	8 (14%)
Guatemala	49	**360**	26 (53%)	23 (47%)	**306**	12 (24%)
Jamaica	58	**360**	33 (57%)	25 (43%)	**306**	26 (45%)
Mexico	80	**360**	54 (68%)	26 (32%)	**306**	42 (53%)
Panama	44	**360**	28 (64%)	16 (36%)	**306**	16 (36%)
Paraguay	28	**360**	22 (79%)	6 (21%)	**306**	19 (68%)
Peru	39	**360**	29 (74%)	10 (26%)	**306**	15 (38%)
Trinidad and Tobago	75	**360**	35 (47%)	40 (53%)	**306**	24 (32%)
Barbados	80	**360**	54 (68%)	26 (33%)	**306**	44 (55%)
**Noodles in Broth- as consumed**	**207**		**169 (82%)**	**38 (18%)**		**114 (55%)**
Argentina	0	**430**	0 (0%)	0 (0%)	**360**	0 (0%)
Brazil	7	**430**	7 (100%)	0 (0%)	**360**	4 (57%)
Chile	12	**430**	12 (100%)	0 (0%)	**360**	11 (92%)
Costa Rica	13	**430**	8 (62%)	5 (38%)	**360**	5 (38%)
Cuba	1	**430**	1 (100%)	0 (0%)	**360**	1 (100%)
Ecuador	4	**430**	1 (25%)	3 (75%)	**360**	1(25%)
Guatemala	30	**430**	15 (50%)	15 (50%)	**360**	8 (27%)
Jamaica	32	**430**	32 (100%)	0 (100%)	**360**	21 (70%)
Mexico	0	**430**	0 (0%)	0 (0%)	**360**	0 (0%)

	n	Regional Target (mg Na/100 g)	% Meeting Regional Target	% Exceeding Regional Target	Lower Target (mg Na/100 g)	% Meeting Lower Target
Panama	27	**430**	24 (89%)	3 (11%)	**360**	20 (74%)
Paraguay	3	**430**	3 (100%)	0 (0%)	**360**	3 (100%)
Peru	55	**430**	50 (91%)	5 (9%)	**360**	30 (55%)
Trinidad and Tobago	23	**430**	16 (70%)	7 (30%)	**360**	10 (43%
Barbados	0	**430**	0 (0%)	0 (0%)	**360**	0 (0%)
**Meats and Cooked, raw and processed Sausages**	**1535**		**1329 (87%)**	**206 (13%)**		**378 (25%)**
Argentina	162	**1210**	143 (88%)	19 (12%)	**690**	20 (12%)
Brazil	162	**1210**	120 (74%)	42 (26%)	**690**	20 (12%)
Chile	164	**1210**	146 (89%)	18 (11%)	**690**	25 (15%)
Costa Rica	60	**1210**	42 (70%)	18 (30%)	**690**	19 (32%)
Cuba	26	**1210**	22 (85%)	4 (15%)	**690**	10 (38%)
Ecuador	193	**1210**	167 (87%)	26 (13%)	**690**	31 (16%)
Guatemala	38	**1210**	31 (82%)	7 (18%)	**690**	6 (16%)
Jamaica	68	**1210**	60 (88%)	8 (12%)	**690**	21 (31%)
Mexico	148	**1210**	138 (93%)	10 (7%)	**690**	38 (26%)
Panama	174	**1210**	155 (89%)	19 (11%)	**690**	74 (43%)
Paraguay	109	**1210**	101 (93%)	8 (7%)	**690**	50 (46%)
Peru	47	**1210**	42 (89%)	5 (11%)	**690**	27 (57%)
Trinidad and Tobago	45	**1210**	44 (98%)	1 (2%)	**690**	15 (33%)
Barbados	139	**1210**	118 (85%)	21 (15%)	**690**	22 (16%)
**Cured dry meats and meats preserved at room temperature**	**320**		**290 (91%)**	**30 (9%)**		**213 (67%)**
Argentina	28	**1900**	20 (72%)	8 (28%)	**1350**	10 (36%)
Brazil	13	**1900**	10 (77%)	3 (23%)	**1350**	4 (31%)

	n	Regional Target (mg Na/100 g)	% Meeting Regional Target	% Exceeding Regional Target	Lower Target (mg Na/100 g)	% Meeting Lower Target
Chile	37	**1900**	34 (92%)	3 (8%)	**1350**	10 (27%)
Costa Rica	0	**1900**	0 (0%)	0 (0%)	**1350**	0 (0%)
Cuba	4	**1900**	3 (75%)	1 (25%)	**1350**	2 (50%)
Ecuador	11	**1900**	9 (82%)	2 (18%)	**1350**	6 (55%)
Guatemala	16	**1900**	14 (88%)	2 (13%)	**1350**	13 (81%)
Jamaica	31	**1900**	31 (100%)	0 (0%)	**1350**	30 (97%)
Mexico	49	**1900**	41 (84%)	8 (16%)	**1350**	28 (57%)
Panama	0	**1900**	0 (0%)	0 (0%)	**1350**	0 (0%)
Paraguay	12	**1900**	12 (100%)	0(0%)	**1350**	8 (67%)
Peru	1	**1900**	1 (100%)	0 (0%)	**1350**	1 (100%)
Trinidad and Tobago	108	**1900**	105 (97%)	3 (3%)	**1350**	100 (93)
Barbados	10	**1900**	10 (100%)	0 (0%)	**1350**	1 (1%)
**Breaded meat and poultry**	**216**		**141 (65%)**	**75 (35%)**		**60 (28%)**
Argentina	22	**735**	22 (100%)	0 (0%)	**470**	6 (27%)
Brazil	25	**735**	9 (36%)	16 (64%)	**470**	2 (8%)
Chile	18	**735**	16 (89%)	2 (11%)	**470**	10 (56%)
Costa Rica	41	**735**	17 (41%)	24(59%)	**470**	4 (10%)
Cuba	4	**735**	4 (100%)	0 (0%)	**470**	4 (100%)
Ecuador	17	**735**	10 (59%)	7 (41%)	**470**	3 (18)
Guatemala	6	**735**	1 (17%)	5 (83%)	**470**	0 (0%)
Jamaica	18	**735**	16 (89%)	2 (11%)	**470**	10 (55%)
Mexico	17	**735**	13 (76%)	4 (24%)	**470**	8 (47%)
Panama	13	**735**	10 (77%)	3 (23%)	**470**	6 (46%)
Paraguay	8	**735**	4 (50%)	4 (50%)	**470**	2 (25%)
Peru	1	**735**	1 (100%)	0 (0%)	**470**	0 (0%)
Trinidad and Tobago	26	**735**	17 (65%)	9 (35%)	**470**	10 (38%)
Barbados	17	**735**	16 (94%)	1 (6%)	**470**	4 (24%)

	n	Regional Target (mg Na/100 g)	% Meeting Regional Target	% Exceeding Regional Target	Lower Target (mg Na/100 g)	% Meeting Lower Target
**Bread**	**1271**		**1053 (83%)**	**218 (17%)**		**434 (34%)**
Argentina	123	**600**	91 (74%)	32 (26%)	**400**	41 (33%)
Brazil	101	**600**	93 (93%)	8 (8%)	**400**	47 (47%)
Chile	106	**600**	99 (93%)	7 (7%)	**400**	48 (45%)
Costa Rica	83	**600**	62 (75%)	21 (25%)	**400**	30 (36%)
Cuba	24	**600**	17 (71%)	7 (29%)	**400**	1 (4%)
Ecuador	106	**600**	97 (92%)	9 (8%)	**400**	38 (36%)
Guatemala	68	**600**	57 (84%)	11 (16%)	**400**	28 (41%)
Jamaica	72	**600**	66 (92%)	6 (8%)	**400**	14 (19%)
Mexico	112	**600**	76 (69%)	36 (31%)	**400**	37 (33%)
Panama	137	**600**	99 (72%)	38 (28%)	**400**	31 (23%)
Paraguay	76	**600**	67 (88%)	9 (12%)	**400**	34 (45%)
Peru	68	**600**	53 (78%)	15 (22%)	**400**	16 (24%)
Trinidad and Tobago	95	**600**	87 (92%)	8 (8%)	**400**	36 (38%)
Barbados	100	**600**	89 (89%)	11 (11%)	**400**	33 (33%)
**Mayonnaise**	**337**		**317 (94%)**	**20 (6%)**		**128 (38%)**
Argentina	18	**1050**	17 (95%)	1 (6%)	**670**	3 (17%)
Brazil	33	**1050**	29 (88%)	4 (12%)	**670**	5 (15%)
Chile	30	**1050**	30 (100%)	0 (0%)	**670**	12 (40%)
Costa Rica	34	**1050**	34 (100%)	0 (0%)	**670**	9 (26%)
Cuba	6	**1050**	6 (100%)	0 (0%)	**670**	5 (83%)
Ecuador	26	**1050**	25 (96%)	1 (4%)	**670**	20 (77%)
Guatemala	29	**1050**	25 (86%)	4 (14%)	**670**	14 (48%)
Jamaica	11	**1050**	11 (100%)	0 (0%)	**670**	7 (64%)
Mexico	37	**1050**	35 (95%)	2 (5%)	**670**	14 (38%)
Panama	25	**1050**	24 (96%)	1 (4%)	**670**	8 (32%)

	n	Regional Target (mg Na/100 g)	% Meeting Regional Target	% Exceeding Regional Target	Lower Target (mg Na/100 g)	% Meeting Lower Target
Paraguay	18	**1050**	17 (94%)	1 (6%)	**670**	1 (6%)
Peru	20	**1050**	18 (90%)	2 (10%)	**670**	8 (40%)
Trinidad and Tobago	20	**1050**	19 (95%)	1 (5%)	**670**	12 (60%)
Barbados	30	**1050**	27 (90%)	3 (10%)	**670**	10 (33%)
**Cookies and Sweet Cookies**	**1560**		**1406 (90%)**	**154 (10%)**		**750 (48%)**
Argentina	98	**485**	94 (96%)	4 (4%)	**265**	54 (55%)
Brazil	110	**485**	110 (100%)	0 (0%)	**265**	67 (61%)
Chile	106	**485**	106 (100%)	0 (0%)	**265**	64 (60%)
Costa Rica	127	**485**	118 (93%)	9 (7%)	**265**	62 (49%)
Cuba	21	**485**	21 (100%)	0 (0%)	**265**	13 (62%)
Ecuador	146	**485**	133 (91%)	13 (9%)	**265**	97 (66%)
Guatemala	111	**485**	94 (85%)	17 (15%)	**265**	55(50%)
Jamaica	115	**485**	103 (90%)	12 (10%)	**265**	30 (26%)
Mexico	93	**485**	85 (91%)	8 (9%)	**265**	53 (57%)
Panama	128	**485**	106 (83%)	22 (17%)	**265**	55 (43%)
Paraguay	116	**485**	114 (98%)	2 (2%)	**265**	66 (57%)
Peru	91	**485**	82 (90%)	9 (10%)	**265**	40 (44%)
Trinidad and Tobago	190	**485**	145 (76%)	45 (24%)	**265**	57 (30%)
Barbados	108	**485**	95 (88%)	13 (12%)	**265**	37 (34%)
**Flavored Cookies and Crackers**	**609**		**591 (97%)**	**18 (3%)**		**362 (59%)**
Argentina	67	**1340**	66 (98%)	1 (2%)	**700**	45 (67%)
Brazil	40	**1340**	38 (95%)	2 (5%)	**700**	33 (83%)
Chile	46	**1340**	45 (98%)	1 (2%)	**700**	37 (80%)
Costa Rica	43	**1340**	38 (88%)	5 (12%)	**700**	14 (33%)
Cuba	8	**1340**	8 (100%)	0 (0%)	**700**	7 (88%)

	n	Regional Target (mg Na/100 g)	% Meeting Regional Target	% Exceeding Regional Target	Lower Target (mg Na/100 g)	% Meeting Lower Target
Ecuador	38	**1340**	38 (100%)	0 (0%)	**700**	26 (64%)
Guatemala	36	**1340**	32 (89%)	4 (11%)	**700**	18 (50%)
Jamaica	56	**1340**	56 (100%)	0 (0%)	**700**	27 (48%)
Mexico	79	**1340**	77 (97%)	2 (3%)	**700**	58 (73%)
Panama	71	**1340**	70 (99%)	1 (1%)	**700**	28 (39%)
Paraguay	32	**1340**	32 (100%)	0 (0%)	**700**	21 (66%)
Peru	38	**1340**	37 (97%)	1 (3%)	**700**	20 (53%)
Trinidad and Tobago	3	**1340**	3 (100%)	0 (0%)	**700**	2 (67%)
Barbados	52	**1340**	51 (98%)	1 (2%)	**700**	26 (50%)
**Cakes**	**1443**		**919 (64%)**	**524 (36%)**		**356 (25%)**
Argentina	127	**400**	89 (70%)	38 (30%)	**205**	31 (24%)
Brazil	154	**400**	104 (68%)	50 (32%)	**205**	43 (28%)
Chile	157	**400**	141 (90%)	16 (10%)	**205**	86 (55%)
Costa Rica	117	**400**	78 (66%)	39 (33%)	**205**	32 (27%)
Cuba	8	**400**	8 (100%)	0 (0%)	**205**	2 (25%)
Ecuador	135	**400**	95 (70%)	40 (30%)	**205**	54 (40%)
Guatemala	96	**400**	48 (50%)	48 (50%)	**205**	18 (19%)
Jamaica	75	**400**	27 (36%)	48 (64%)	**205**	4 (5%)
Mexico	121	**400**	84 (69%)	37 (31%)	**205**	22 (18%)
Panama	129	**400**	74 (61%)	55 (43%)	**205**	20 (16%)
Paraguay	35	**400**	11 (31%)	24 (69%)	**205**	5 (14%)
Peru	33	**400**	19 (58%)	14 (42%)	**205**	9 (27%)
Trinidad and Tobago	127	**400**	79 (62%)	48 (38%)	**205**	15 (12%)
Barbados	129	**400**	62 (48%)	67 (52%)	**205**	15 (12%)
**Breakfast Cereals**	**1457**		**1326 (91%)**	**131 (9%)**		**1114 (76%)**
Argentina	88	**630**	76 (87%)	12 (14)%	**500**	63 (72%)

	n	Regional Target (mg Na/100 g)	% Meeting Regional Target	% Exceeding Regional Target	Lower Target (mg Na/100 g)	% Meeting Lower Target
Brazil	100	**630**	94 (94%)	6 (6%)	**500**	92 (92%)
Chile	109	**630**	108 (99%)	1 (1%)	**500**	101 (93%)
Costa Rica	115	**630**	99 (86%)	16 (14%)	**500**	78 (68%)
Cuba	23	**630**	19 (83%)	4 (17%)	**500**	14 (61%)
Ecuador	100	**630**	93 (93%)	7 (7%)	**500**	87 (87%)
Guatemala	100	**630**	89 (89%)	11 (11%)	**500**	63 (63%)
Jamaica	114	**630**	94 (82%)	20 (18%)	**500**	72 (63%)
Mexico	104	**630**	101 (97%)	3 (3%)	**500**	82 (79%)
Panama	190	**630**	179 (94%)	11 (6%)	**500**	143 (75%)
Paraguay	98	**630**	93 (95%)	5 (5%)	**500**	86 (88%)
Peru	91	**630**	82 (90%)	9 (10%)	**500**	75 (82%)
Trinidad and Tobago	112	**630**	97 (87%)	15 (13%)	**500**	78 (70%)
Barbados	113	**630**	102 (90%)	11 (10%)	**500**	80 (71%)
**Butter**	**507**		**428 (84%)**	**79 (16%)**		**186 (37%)**
Argentina	23	**800**	23 (100%)	0 (0%)	**500**	19 (83%)
Brazil	50	**800**	36 (72%)	14 (28%)	**500**	11 (22%)
Chile	50	**800**	47 (94%)	3 (6%)	**500**	17 (34%)
Costa Rica	43	**800**	35 (81%)	8 (19%)	**500**	17 (40%)
Cuba	1	**800**	1 (100%)	0 (0%)	**500**	0 (0%)
Ecuador	32	**800**	22 (69%)	10 (31%)	**500**	15 (47%)
Guatemala	68	**800**	58 (85%)	10 (15%)	**500**	21 (31%)
Jamaica	30	**800**	26 (87%)	4 (13%)	**500**	6 (20%)
Mexico	46	**800**	44 (96%)	2 (4%)	**500**	34 (74%)
Panama	51	**800**	45 (88%)	6 (12%)	**500**	10 (20%)
Paraguay	17	**800**	14 (82%)	3 (18%)	**500**	9 (53%)
Peru	18	**800**	10 (56%)	8 (44%)	**500**	6 (33%)
Trinidad and Tobago	28	**800**	20 (71%)	8 (29%)	**500**	6 (21%)

	n	Regional Target (mg Na/100 g)	% Meeting Regional Target	% Exceeding Regional Target	Lower Target (mg Na/100 g)	% Meeting Lower Target
Barbados	50	**800**	47 (94%)	3 (6%)	**500**	15 (30%)
**Snacks**	**2235**		**1674 (75%)**	**561 (25%)**		**865 (39%)**
Argentina	123	**900**	93 (76%)	30 (25%)	**530**	45 (37%)
Brazil	198	**900**	130 (66%)	68 (34%)	**530**	54 (27%)
Chile	132	**900**	126 (95%)	6 (5%)	**530**	85 (64%)
Costa Rica	206	**900**	149 (72%)	57 (28%)	**530**	77 (37%)
Cuba	14	**900**	13 (93%)	1 (7%)	**530**	4 (29%)
Ecuador	187	**900**	157 (84%)	30 (16%)	**530**	102 (55%)
Guatemala	181	**900**	131 (72%)	50 (28%)	**530**	68 (38%)
Jamaica	147	**900**	108 (73%)	39 (27%)	**530**	62 (42%)
Mexico	211	**900**	150 (71%)	61 (29%)	**530**	57 (27%)
Panama	207	**900**	139 (67%)	68 (33%)	**530**	74 (38%)
Paraguay	159	**900**	115 (72%)	44 (28%)	**530**	60 (38%)
Peru	116	**900**	102 (88%)	14 (12%)	**530**	55 (47%)
Trinidad and Tobago	152	**900**	111 (73%)	41 (27%)	**530**	58 (38%)
Barbados	202	**900**	150 (74%)	52 (26%)	**530**	64 (32%)
**Pasta and Noodles -dry, uncooked**	**696**		**681 (98%)**	**15 (2%)**		**609 (88%)**
Argentina	96	**1921**	96 (100%)	0 (0%)	**1333**	93 (97%)
Brazil	50	**1921**	49 (98%)	1 (2%)	**1333**	12 (24%)
Chile	7	**1921**	7 (100%)	0 (0%)	**1333**	4 (57%)
Costa Rica	48	**1921**	45 (94%)	3 (6%)	**1333**	41 (85%)
Cuba	30	**1921**	30 (100%)	0 (0%)	**1333**	30 (100%)
Ecuador	50	**1921**	47 (94%)	3 (6%)	**1333**	47 (94%)
Guatemala	66	**1921**	66 (100%)	0 (0%)	**1333**	63 (96%)
Jamaica	19	**1921**	19 (100%)	0 (0%)	**1333**	18 (95%)
Mexico	24	**1921**	24 (100%)	0 (0%)	**1333**	20 (83%)

	n	Regional Target (mg Na/100 g)	% Meeting Regional Target	% Exceeding Regional Target	Lower Target (mg Na/100 g)	% Meeting Lower Target
Panama	18	**1921**	15 (83%)	3 (17%)	**1333**	14 (78%)
Paraguay	7	**1921**	7 (100%)	0 (0%)	**1333**	4 (57%)
Peru	73	**1921**	72 (99%)	1 (1%)	**1333**	72 (97%)
Trinidad and Tobago	46	**1921**	45 (98%)	1 (2%)	**1333**	44 (96%)
Barbados	49	**1921**	47 (96%)	2 (4%)	**1333**	42 (86%)
**Pasta and Noodles- as consumed**	**153**		**140 (92%)**	**13 (8%)**		**134 (88%)**
Argentina	0	**640**	0 (0%)	0 (0%)	**440**	0 (0%)
Brazil	1	**640**	1 (100%)	0 (0%)	**440**	1 (100%)
Chile	50	**640**	46 (92%)	4(8%)	**440**	45 (90%)
Costa Rica	0	**640**	0(0%)	0(0%)	**440**	0 (0%)
Cuba	0	**640**	0 (0%)	0 (0%)	**440**	0 (0%)
Ecuador	0	**640**	0(0%)	0 (0%)	**440**	0 (0%)
Guatemala	0	**640**	0 (0%)	0 (0%)	**440**	0 (0%)
Jamaica	12	**640**	7 (58%)	5 (42%)	**440**	7(58%)
Mexico	22	**640**	21 (95%)	1(5%)	**440**	20 (91%)
Pasta and Noodles, as consumed	46	**640**	43 (93%)	3 (7%)	**440**	40 (87%)
Paraguay	0	**640**	0(0%)	0(0%)	**440**	0(0%)
Peru	0	**640**	0(0%)	0(0%)	**440**	0(0%)
Trinidad and Tobago	14	**640**	14 (100%)	0(0%)	**440**	13 (93%)
Barbados	8	**640**	8 (100%)	0(0%)	**440**	8 (100%)
**Seasonings for Side and Main Dishes**	**390**		**375 (96%)**	**15 (4%)**		**286 (73%)**
Argentina	22	**33100**	22 (100%)	0 (0%)	**9100**	18 (82%)
Brazil	2	**33100**	2 (100%)	0 (0%)	**9100**	0 (0%)
Chile	63	**33100**	63 (100%)	0(0%)	**9100**	45 (71%)

	n	Regional Target (mg Na/100 g)	% Meeting Regional Target	% Exceeding Regional Target	Lower Target (mg Na/100 g)	% Meeting Lower Target
Costa Rica	44	**33100**	44(100%)	0(0%)	**9100**	24 (55%)
Cuba	12	**33100**	12(100%)	0(0%)	**9100**	11 (92%)
Ecuador	8	**33100**	8(100%)	0(0%)	**9100**	8(100%)
Guatemala	6	**33100**	6(100%)	0(0%)	**9100**	5 (83%)
Jamaica	13	**33100**	13(100%)	0(0%)	**9100**	12 (92%)
Mexico	31	**33100**	31(100%)	0(0%)	**9100**	16 (52%)
Panama	41	**33100**	41(100%)	0(0%)	**9100**	41(100%)
Paraguay	28	**33100**	24(86%)	4 (14%)	**9100**	5 (18%)
Peru	51	**33100**	51(100%)	0(0%)	**9100**	44 (86%)
Trinidad and Tobago	51	**33100**	40 (78%)	11 (22%)	**9100**	40 (78%)
Barbados	18	**33100**	18 (100%)	0(0%)	**9100**	17 (94%)
**Meat and Fish Seasonings**	**435**		**385 (89%)**	**50 (11%)**		**381 (88%)**
Argentina	26	**23000**	23 (0%)	3 (12%)	**21775**	23 (89%)
Brazil	48	**23000**	29 (60%)	19 (40%)	**21775**	27 (57%)
Chile	2	**23000**	2(100%)	0 (0%)	**21775**	2(100%)
Costa Rica	15	**23000**	15(100%)	0(0%)	**21775**	15(100%)
Cuba	4	**23000**	4(100%)	0(0%)	**21775**	4(100%)
Ecuador	54	**23000**	51 (94%)	3(6%)	**21775**	51 (94%)
Guatemala	34	**23000**	34 (100%)	0(0%)	**21775**	34 (100%)
Jamaica	46	**23000**	38 (83%)	8 (17%)	**21775**	37 (80%)
Mexico	61	**23000**	51 (84%)	10 (16%)	**21775**	50 (82%)
Panama	38	**23000**	37 (97%)	1 (3%)	**21775**	37 (97%)
Paraguay	6	**23000**	3 (50%)	3 (50%)	**21775**	3(50%)
Peru	11	**23000**	11(100%)	0(0%)	**21775**	11(100%)
Trinidad and Tobago	20	**23000**	19 (95%)	1 (5%)	**21775**	19 (95%)
Barbados	70	**23000**	68 (97%)	2 (3%)	**21775**	68 (97%)

	n	Regional Target (mg Na/100 g)	% Meeting Regional Target	% Exceeding Regional Target	Lower Target (mg Na/100 g)	% Meeting Lower Target
**Bouillon Cubes and Powders, as consumed**	**258**		**159 (62%)**	**99 (38%)**		**96 (37%)**
Argentina	23	**20500**	14 (62%)	9 (39%)	**18000**	7 (31%)
Brazil	57	**20500**	34 (60%)	23 (40%)	**18000**	23 (41%)
Chile	20	**20500**	12 (60%)	8 (40%)	**18000**	6 (30%)
Costa Rica	26	**20500**	16 (62%)	10 (38%)	**18000**	10(38%)
Cuba	7	**20500**	6 (86%)	1 (14%)	**18000**	4 (57%)
Ecuador	14	**20500**	13 (93%)	1 (7%)	**18000**	10 (71%)
Guatemala	10	**20500**	7(70%)	3 (30%)	**18000**	4 (10%)
Jamaica	7	**20500**	6 (86%)	1 (14%)	**18000**	4(57%)
Mexico	32	**20500**	18 (56%)	14 (44%)	**18000**	9 (28%)
Panama	0	**20500**	0(0%)	0(0%)	**18000**	0(0%)
Paraguay	6	**20500**	5 (83%)	1(17%)	**18000**	1 (17%)
Peru	24	**20500**	8 (33%)	16 (67%)	**18000**	5(21%)
Trinidad and Tobago	6	**20500**	3 (50%)	3 (50%)	**18000**	3 (50%)
Barbados	26	**20500**	17 (65%)	9 (34%)	**18000**	10 (38%)

Data presented as n (%).

Table S5. Detailed assessment of the proportion of foods meeting the Regional Sodium Targets by Country

	n	Regional Target (mg Na/100 g)	% Meeting Regional Target	% Exceeding Regional Target	Lower Target (mg Na/100 g)	% Meeting Lower Target
**Argentina**	**1125**		**940 (84%)**	**185 (16%)**		**508 (45%)**
Wet and Dry Soups	79	**360**	51 (65%)	28 (35%)	**306**	30 (38%)
Noodles in Broth	0	**430**	0 (0%)	0 (0%)	**360**	0 (0%)
Meats and Sausages	162	**1210**	143 (88%)	19 (12%)	**690**	20 (12%)
Cured and Preserved Meats	28	**1900**	20 (72%)	8 (28%)	**1350**	10 (36%)
Breaded meat and poultry	22	**735**	22 (100%)	0 (0%)	**470**	6 (27%)
Bread	123	**600**	91 (74%)	32 (26%)	**400**	41 (33%)
Mayonnaise	18	**1050**	17 (95%)	1 (6%)	**670**	3 (17%)
Cookies and Sweet Cookies	98	**485**	94 (96%)	4 (4%)	**265**	54 (55%)
Flavored Cookies and Crackers	67	**1340**	66 (98%)	1 (2%)	**700**	45 (67%)
Cakes	127	**400**	89 (70%)	38 (30%)	**205**	31 (24%)
Breakfast Cereals	88	**630**	76 (87%)	12 (14)%	**500**	63 (72%)
Butter	23	**800**	23 (100%)	0 (0%)	**500**	19 (83%)
Snacks	123	**900**	93 (76%)	30 (25%)	**530**	45 (37%)
Pasta and Noodles, uncooked	96	**1921**	96 (100%)	0 (0%)	**1333**	93 (97%)
Pasta and Noodles, as consumed	0	**640**	0 (0%)	0 (0%)	**440**	0 (0%)
Seasonings for Side and Main Dishes	22	**33100**	22 (100%)	0 (0%)	**9100**	18 (82%)
Meat and Fish Seasonings	26	**23000**	23 (0%)	3 (12%)	**21775**	23 (89%)
Bouillon Cubes and Powders	23	**20500**	14 (62%)	9 (39%)	**18000**	7 (31%)
**Brazil**	**1224**		**944 (77%)**	**280 (23%)**		**483 (39%)**
Wet and Dry Soups	73	**360**	49 (67%)	24 (33%)	**306**	38 (52%)
Noodles in Broth	7	**430**	7 (100%)	0 (0%)	**360**	4 (57%)
Meats and Sausages	162	**1210**	120 (74%)	42 (26%)	**690**	20 (12%)
Cured and Preserved Meats	13	**1900**	10 (77%)	3 (23%)	**1350**	4 (31%)
Breaded meat and poultry	25	**735**	9 (36%)	16 (64%)	**470**	2 (8%)
Bread	101	**600**	93 (93%)	8 (8%)	**400**	47 (47%)
Mayonnaise	33	**1050**	29 (88%)	4 (12%)	**670**	5 (15%)
Cookies and Sweet Cookies	110	**485**	110 (100%)	0 (0%)	**265**	67 (61%)
Flavored Cookies and Crackers	40	**1340**	38 (95%)	2 (5%)	**700**	33 (83%)

	n	Regional Target (mg Na/100 g)	% Meeting Regional Target	% Exceeding Regional Target	Lower Target (mg Na/100 g)	% Meeting Lower Target
Cakes	154	**400**	104 (68%)	50 (32%)	**205**	43 (28%)
Breakfast Cereals	100	**630**	94 (94%)	6 (6%)	**500**	92 (92%)
Butter	50	**800**	36 (72%)	14 (28%)	**500**	11 (22%)
Snacks	198	**900**	130 (66%)	68 (34%)	**530**	54 (27%)
Pasta and Noodles, uncooked	50	**1921**	49 (98%)	1 (2%)	**1333**	12 (24%)
Pasta and Noodles, as consumed	1	**640**	1 (100%)	0 (0%)	**440**	1 (100%)
Seasonings for Side and Main Dishes	2	**33100**	2 (100%)	0 (0%)	**9100**	0 (0%)
Meat and Fish Seasonings	48	**23000**	29 (60%)	19 (40%)	**21775**	27 (57%)
Bouillon Cubes and Powders	57	**20500**	34 (60%)	23 (40%)	**18000**	23 (41%)
**Chile**	**1178**		**1087 (92%)**	**91 (8%)**		**652 (55%)**
Wet and Dry Soups	69	**360**	47 (68%)	22 (32%)	**306**	44 (64%)
Noodles in Broth	12	**430**	12(100%)	0 (0%)	**360**	11 (92%)
Meats and Sausages	164	**1210**	146 (89%)	18 (11%)	**690**	25 (15%)
Cured and Preserved Meats	37	**1900**	34 (92%)	3 (8%)	**1350**	10 (27%)
Breaded meat and poultry	18	**735**	16 (89%)	2 (11%)	**470**	10 (56%)
Bread	106	**600**	99 (93%)	7 (7%)	**400**	48 (46%)
Mayonnaise	30	**1050**	30 (100%)	0 (0%)	**670**	12 (40%)
Cookies and Sweet Cookies	106	**485**	106 (100%)	0 (0%)	**265**	64 (61%)
Flavored Cookies and Crackers	46	**1340**	45 (98%)	1 (2%)	**700**	37 (81%)
Cakes	157	**400**	141 (90%)	16 (10%)	**205**	86 (55%)
Breakfast Cereals	109	**630**	108 (99%)	1 (1%)	**500**	101 (93%)
Butter	50	**800**	47 (94%)	3 (6%)	**500**	17 (34%)
Snacks	132	**900**	126 (95%)	6 (5%)	**530**	85 (65%)
Pasta and Noodles, uncooked	7	**1921**	7 (100%)	0 (0%)	**1333**	4 (57%)
Pasta and Noodles, as consumed	50	**640**	46 (92%)	4 (8%)	**440**	45 (90%)
Seasonings for Side and Main Dishes	63	**33100**	63 (100%)	0 (0%)	**9100**	45 (72%)
Meat and Fish Seasonings	2	**23000**	2 (100%)	0 (0%)	**21775**	2 (100%)
Bouillon Cubes and Powders	20	**20500**	12 (60%)	8 (40%)	**18000**	6 (30%)

	n	Regional Target (mg Na/100 g)	% Meeting Regional Target	% Exceeding Regional Target	Lower Target (mg Na/100 g)	% Meeting Lower Target
**Costa Rica**	**1086**		**834 (77%)**	**252 (23%)**		**460(43%)**
Wet and Dry Soups	71	**360**	34 (48%)	37 (52%)	**306**	23 (32%)
Noodles in Broth	13	**430**	8 (61%)	5 (39%)	**360**	5 (39%)
Meats and Sausages	60	**1210**	42 (70%)	18 (30%)	**690**	19 (32%)
Cured and Preserved Meats	0	**1900**	0 (0%)	0 (0%)	**1350**	0 (0%)
Breaded meat and poultry	41	**735**	17 (41%)	24 (59%)	**470**	4 (10%)
Bread	83	**600**	62 (75%)	21 (25%)	**400**	30 (36%)
Mayonnaise	34	**1050**	34 (100%)	0 (0%)	**670**	9 (27%)
Cookies and Sweet Cookies	127	**485**	118 (93%)	9 (7%)	**265**	62 (49%)
Flavored Cookies and Crackers	43	**1340**	38 (88%)	5 (12%)	**700**	14 (32%)
Cakes	117	**400**	78 (67%)	39 (33%)	**205**	32 (28%)
Breakfast Cereals	115	**630**	99 (86%)	16 (14%)	**500**	78 (68%)
Butter	43	**800**	35 (81%)	8 (19%)	**500**	17 (40%)
Snacks	206	**900**	149 (72%)	57 (28%)	**530**	77 (38%)
Pasta and Noodles, uncooked	48	**1921**	45 (94%)	3 (6%)	**1333**	41 (85%)
Pasta and Noodles, as consumed	0	**640**	0 (0%)	0 (0%)	**440**	0 (0%)
Seasonings for Side and Main Dishes	44	**33100**	44 (100%)	0 (0%)	**9100**	24 (56%)
Meat and Fish Seasonings	15	**23000**	15 (100%)	0 (0%)	**21775**	15 (100%)
Bouillon Cubes and Powders	26	**20500**	16 (62%)	10 (38%)	**18000**	10 (39%)
**Cuba**	**209**		**188 (90%)**	**21(10%)**		**124 (59%)**
Wet and Dry Soups	16	**360**	13 (81%)	3 (19%)	**306**	12 (75%)
Noodles in Broth	1	**430**	1 (100%)	0 (0%)	**360**	1 (100%)
Meats and Sausages	26	**1210**	22 (85%)	4 (15%)	**690**	10 (39%)
Cured and Preserved Meats	4	**1900**	3 (75%)	1 (25%)	**1350**	2 (50%)
Breaded meat and poultry	4	**735**	4 (100%)	0 (0%)	**470**	4 (100%)
Bread	24	**600**	17 (71%)	7 (29%)	**400**	1 (4%)
Mayonnaise	6	**1050**	6 (100%)	0 (0%)	**670**	5 (84%)
Cookies and Sweet Cookies	21	**485**	21 (100%)	0 (0%)	**265**	13 (62%)
Flavored Cookies and Crackers	8	**1340**	8 (100%)	0 (0%)	**700**	7 (88%)
Cakes	8	**400**	8 (100%)	0 (0%)	**205**	2 (25%)
Breakfast Cereals	23	**630**	19 (83%)	4 (17%)	**500**	14 (61%)

	n	Regional Target (mg Na/100 g)	% Meeting Regional Target	% Exceeding Regional Target	Lower Target (mg Na/100 g)	% Meeting Lower Target
Butter	1	**800**	1 (100%)	0 (0%)	**500**	0 (0%)
Snacks	14	**900**	13 (93%)	1 (7%)	**530**	4 (29%)
Pasta and Noodles, uncooked	30	**1921**	30 (100%)	0 (0%)	**1333**	30 (100%)
Pasta and Noodles, as consumed	0	**640**	0 (0%)	0 (0%)	**440**	0 (0%)
Seasonings for Side and Main Dishes	12	**33100**	12 (100%)	0 (0%)	**9100**	11 (92%)
Meat and Fish Seasonings	4	**23000**	4(100%)	0 (0%)	**21775**	4 (100%)
Bouillon Cubes and Powders	7	**20500**	6 (86%)	1 (14%)	**18000**	4 (57%)
**Ecuador**	**1177**		**976 (83%)**	**201 (17%)**		**604 (51%)**
Wet and Dry Soups	56	**360**	10 (18%)	46 (82%)	**306**	8 (14%)
Noodles in Broth	4	**430**	1 (25%)	3 (75%)	**360**	1 (25%)
Meats and Sausages	193	**1210**	167 (87%)	26 (13%)	**690**	31 (16%)
Cured and Preserved Meats	11	**1900**	9 (82%)	2 (18%)	**1350**	6 (55%)
Breaded meat and poultry	17	**735**	10 (59%)	7 (41%)	**470**	3 (18%)
Bread	106	**600**	97 (92%)	9 (9%)	**400**	38 (36%)
Mayonnaise	26	**1050**	25 (96%)	1 (4%)	**670**	20 (77%)
Cookies and Sweet Cookies	146	**485**	133 (92%)	13 (9%)	**265**	97 (67%)
Flavored Cookies and Crackers	38	**1340**	38 (100%)	0 (0%)	**700**	26 (69%)
Cakes	135	**400**	95 (70%)	40 (30%)	**205**	54 (40%)
Breakfast Cereals	100	**630**	93 (93%)	7 (7%)	**500**	87 (87%)
Butter	32	**800**	22 (69%)	10 (31%)	**500**	15 (47%)
Snacks	187	**900**	157 (84%)	30 (16%)	**530**	102 (55%)
Pasta and Noodles, uncooked	50	**1921**	47 (94%)	3 (6%)	**1333**	47 (94%)
Pasta and Noodles, as consumed	0	**640**	0 (0%)	0 (0%)	**440**	0 (0%)
Seasonings for Side and Main Dishes	8	**33100**	8 (100%)	0 (0%)	**9100**	8 (100%)
Meat and Fish Seasonings	54	**23000**	51 (94%)	3 (6%)	**21775**	51 (95%)
Bouillon Cubes and Powders	14	**20500**	13 (93%)	1 (7%)	**18000**	10 (72%)
**Guatemala**	**944**		**734 (78%)**	**207 (22%)**		**430 (46%)**
Wet and Dry Soups	49	**360**	26 (53%)	23 (47%)	**306**	12 (25%)
Noodles in Broth	30	**430**	15 (50%)	15 (50%)	**360**	8 (27%)

	n	Regional Target (mg Na/100 g)	% Meeting Regional Target	% Exceeding Regional Target	Lower Target (mg Na/100 g)	% Meeting Lower Target
Meats and Sausages	38	**1210**	31 (82%)	7 (18%)	**690**	6 (16%)
Cured and Preserved Meats	16	**1900**	14 (87%)	2 (13%)	**1350**	13 (81%)
Breaded meat and poultry	6	**735**	1 (17%)	5 (83%)	**470**	0 (0%)
Bread	68	**600**	57 (84%)	11 (16%)	**400**	28 (41%)
Mayonnaise	29	**1050**	25 (86%)	4 (14%)	**670**	14 (48%)
Cookies and Sweet Cookies	111	**485**	94 (85%)	17 (15%)	**265**	55 (50%)
Flavored Cookies and Crackers	36	**1340**	32 (89%)	4 (11%)	**700**	18 (50%)
Cakes	96	**400**	48 (50%)	48 (50%)	**205**	18 (19%)
Breakfast Cereals	100	**630**	89 (89%)	11 (11%)	**500**	63 (63%)
Butter	68	**800**	58 (86%)	10 (15%)	**500**	21 (31%)
Snacks	181	**900**	131 (73%)	50 (28%)	**530**	68 (38%)
Pasta and Noodles, uncooked	66	**1921**	66 (100%)	0 (0%)	**1333**	63 (96%)
Pasta and Noodles, as consumed	0	**640**	0 (0%)	0 (0%)	**440**	0 (0%)
Seasonings for Side and Main Dishes	6	**33100**	6 (100%)	0 (0%)	**9100**	5 (84%)
Meat and Fish Seasonings	34	**23000**	34 (100%)	0 (0%)	**21775**	34 (100%)
Bouillon Cubes and Powders	10	**20500**	7 (70%)	3 (30%)	**18000**	4 (40%)
**Jamaica**	**907**		**731 (81%)**	**176 (19%)**		**399 (44%)**
Wet and Dry Soups	58	**360**	33 (57%)	25 (43%)	**306**	26 (45%)
Noodles in Broth	32	**430**	32 (100%)	0 (0%)	**360**	21 (66%)
Meats and Sausages	68	**1210**	60 (88%)	8 (12%)	**690**	21 (31%)
Cured and Preserved Meats	31	**1900**	31 (100%)	0 (0%)	**1350**	30 (97%)
Breaded meat and poultry	1	**735**	1 (100%)	0 (0%)	**470**	1 (100%)
Bread	72	**600**	66 (92%)	6 (8%)	**400**	14 (20%)
Mayonnaise	11	**1050**	11 (100%)	0 (0%)	**670**	7 (64%)
Cookies and Sweet Cookies	115	**485**	103 (90%)	12 (10%)	**265**	30 (26%)
Flavored Cookies and Crackers	56	**1340**	56 (100%)	0 (0%)	**700**	27 (48%)
Cakes	75	**400**	27 (36%)	48 (64%)	**205**	4 (6%)
Breakfast Cereals	114	**630**	94 (82%)	20 (18%)	**500**	72 (63%)
Butter	30	**800**	26 (87%)	4 (13%)	**500**	6 (20%)
Snacks	147	**900**	108 (74%)	39 (27%)	**530**	62 (42%)

	n	Regional Target (mg Na/100 g)	% Meeting Regional Target	% Exceeding Regional Target	Lower Target (mg Na/100 g)	% Meeting Lower Target
Pasta and Noodles, uncooked	19	**1921**	19 (100%)	0 (0%)	**1333**	18 (95%)
Pasta and Noodles, as consumed	12	**640**	7 (58%)	5 (42%)	**440**	7 (58%)
Seasonings for Side and Main Dishes	13	**33100**	13 (100%)	0 (0%)	**9100**	12 (93%)
Meat and Fish Seasonings	46	**23000**	38 (83%)	8 (18%)	**21775**	37 (81%)
Bouillon Cubes and Powders	7	**20500**	6 (86%)	1 (14%)	**18000**	4 (57%)
**Mexico**	**1267**		**1043 (82%)**	**224 (18%)**		**588 (46%)**
Wet and Dry Soups	80	**360**	54 (68%)	26 (32%)	**306**	42 (53%)
Noodles in Broth	0	**430**	0 (0%)	0 (0%)	**360**	0 (0%)
Meats and Sausages	148	**1210**	138 (93%)	10 (7%)	**690**	38 (26%)
Cured and Preserved Meats	49	**1900**	41 (84%)	8 (16%)	**1350**	28 (57%)
Breaded meat and poultry	17	**735**	13 (76%)	4 (24%)	**470**	8 (47%)
Bread	112	**600**	76 (68%)	36 (32%)	**400**	37 (33%)
Mayonnaise	37	**1050**	35 (95%)	2 (5%)	**670**	14 (38%)
Cookies and Sweet Cookies	93	**485**	85 (91%)	8 (9%)	**265**	53 (57%)
Flavored Cookies and Crackers	79	**1340**	77 (97%)	2 (3%)	**700**	58 (74%)
Cakes	121	**400**	84 (69%)	37 (31%)	**205**	22 (18%)
Breakfast Cereals	104	**630**	101 (97%)	3 (3%)	**500**	82 (79%)
Butter	46	**800**	44 (96%)	2 (4%)	**500**	34 (74%)
Snacks	211	**900**	150 (71%)	61 (29%)	**530**	57 (27%)
Pasta and Noodles, uncooked	24	**1921**	24 (100%)	0 (0%)	**1333**	20 (83%)
Pasta and Noodles, as consumed	22	**640**	21 (96%)	1 (5%)	**440**	20 (91%)
Seasonings for Side and Main Dishes	31	**33100**	31 (100%)	0 (0%)	**9100**	16 (52%)
Meat and Fish Seasonings	61	**23000**	51 (84%)	10 (16%)	**21775**	50 (82%)
Bouillon Cubes and Powders	32	**20500**	18 (56%)	14 (44%)	**18000**	9 (28%)
**Panama**	**1339**		**1089 (81%)**	**250 (19%)**		**607 (45%)**
Wet and Dry Soups	44	**360**	28 (64%)	16 (36%)	**306**	16 (36%)
Noodles in Broth	27	**430**	24 (89%)	3 (11%)	**360**	20 (74%)
Meats and Sausages	174	**1210**	155 (89%)	19 (11%)	**690**	74 (43%)

	n	Regional Target (mg Na/100 g)	% Meeting Regional Target	% Exceeding Regional Target	Lower Target (mg Na/100 g)	% Meeting Lower Target
Cured and Preserved Meats	0	**1900**	0 (0%)	0 (0%)	**1350**	0 (0%)
Breaded meat and poultry	13	**735**	10 (77%)	3 (23%)	**470**	6 (46%)
Bread	137	**600**	99 (72%)	38 (28%)	**400**	31 (23%)
Mayonnaise	25	**1050**	24 (96%)	1 (4%)	**670**	8 (32%)
Cookies and Sweet Cookies	128	**485**	106 (83%)	22 (17%)	**265**	55 (43%)
Flavored Cookies and Crackers	71	**1340**	70 (99%)	1 (1%)	**700**	28 (40%)
Cakes	129	**400**	74 (57%)	55 (43%)	**205**	20 (16%)
Breakfast Cereals	190	**630**	179 (94%)	11 (6%)	**500**	143 (75%)
Butter	51	**800**	45 (88%)	6 (12%)	**500**	10 (20%)
Snacks	207	**900**	139 (67%)	68 (33%)	**530**	74 (36%)
Pasta and Noodles, uncooked	18	**1921**	15 (83%)	3 (17%)	**1333**	14 (78%)
Pasta and Noodles, as consumed	46	**640**	43 (93%)	3 (7%)	**440**	40 (87%)
Seasonings for Side and Main Dishes	41	**33100**	41 (100%)	0 (0%)	**9100**	41 (100%)
Meat and Fish Seasonings	38	**23000**	37 (97%)	1 (3%)	**21775**	37 (98%)
Bouillon Cubes and Powders	0	**20500**	0 (0%)	0 (0%)	**18000**	0 (0%)
**Paraguay**	**871**		**756 (87%)**	**115 (13%)**		**482 (55%)**
Wet and Dry Soups	28	**360**	22 (79%)	6 (21%)	**306**	19 (68%)
Noodles in Broth	3	**430**	3 (100%)	0 (0%)	**360**	3 (100%)
Meats and Sausages	109	**1210**	101 (93%)	8 (7%)	**690**	50 (46%)
Cured and Preserved Meats	12	**1900**	12 (100%)	0 (0%)	**1350**	8 (67%)
Breaded meat and poultry	8	**735**	4 (50%)	4 (50%)	**470**	2 (25%)
Bread	76	**600**	67 (88%)	9 (12%)	**400**	34 (45%)
Mayonnaise	18	**1050**	17 (94%)	1 (6%)	**670**	1 (6%)
Cookies and Sweet Cookies	116	**485**	114 (98%)	2 (2%)	**265**	66 (57%)
Flavored Cookies and Crackers	32	**1340**	32 (100%)	0 (0%)	**700**	21 (66%)
Cakes	35	**400**	11 (31%)	24 (69%)	**205**	5 (15%)
Breakfast Cereals	98	**630**	93 (95%)	5 (5%)	**500**	86 (88%)
Butter	17	**800**	14 (82%)	3 (18%)	**500**	9 (53%)
Snacks	159	**900**	115 (72%)	44 (28%)	**530**	60 (38%)
Pasta and Noodles, uncooked	120	**1921**	119 (99%)	1 (1%)	**1333**	109 (91%)

	n	Regional Target (mg Na/100 g)	% Meeting Regional Target	% Exceeding Regional Target	Lower Target (mg Na/100 g)	% Meeting Lower Target
Pasta and Noodles, as consumed	0	**640**	0 (0%)	0 (0%)	**440**	0 (0%)
Seasonings for Side and Main Dishes	28	**33100**	24 (86%)	4 (14%)	**9100**	5 (18%)
Meat and Fish Seasonings	6	**23000**	3 (50%)	3 (50%)	**21775**	3 (50%)
Bouillon Cubes and Powders	6	**20500**	5 (83%)	1 (17%)	**18000**	1 (17%)
**Peru**	**777**		**668 (86%)**	**109 (14%)**		**434 (56%)**
Wet and Dry Soups	39	**360**	29 (74%)	10 (26%)	**306**	15 (39%)
Noodles in Broth	55	**430**	50 (91%)	5 (9%)	**360**	30 (55%)
Meats and Sausages	47	**1210**	42 (89%)	5 (11%)	**690**	27 (58%)
Cured and Preserved Meats	1	**1900**	1 (100%)	0 (0%)	**1350**	1 (100%)
Breaded meat and poultry	1	**735**	1 (100%)	0 (0%)	**470**	0 (0%)
Bread	68	**600**	53 (78%)	15 (22%)	**400**	16 (24%)
Mayonnaise	20	**1050**	18 (90%)	2 (10%)	**670**	8 (40%)
Cookies and Sweet Cookies	91	**485**	82 (90%)	9 (10%)	**265**	40 (44%)
Flavored Cookies and Crackers	38	**1340**	37 (97%)	1 (3%)	**700**	20 (53%)
Cakes	33	**400**	19 (57%)	14 (43%)	**205**	9 (27%)
Breakfast Cereals	91	**630**	82 (90%)	9 (10%)	**500**	75 (83%)
Butter	18	**800**	10 (56%)	8 (44%)	**500**	6 (34%)
Snacks	116	**900**	102 (88%)	14 (12%)	**530**	55 (48%)
Pasta and Noodles, uncooked	73	**1921**	72 (99%)	1 (1%)	**1333**	72 (99%)
Pasta and Noodles, as consumed	0	**640**	0 (0%)	0 (0%)	**440**	0 (0%)
Seasonings for Side and Main Dishes	51	**33100**	51 (100%)	0 (0%)	**9100**	44 (87%)
Meat and Fish Seasonings	11	**23000**	11 (100%)	0 (0%)	**21775**	11 (100%)
Bouillon Cubes and Powders	24	**20500**	8 (33%)	16 (67%)	**18000**	5 (21%)
**Trinidad and Tobago**	**1141**		**899 (79%)**	**242 (21%)**		**542 (48%)**
Wet and Dry Soups	75	**360**	35 (47%)	40 (53%)	**306**	24 (32%)
Noodles in Broth	23	**430**	16 (70%)	7 (30%)	**360**	10 (44%)
Meats and Sausages	45	**1210**	44 (98%)	1 (2%)	**690**	15 (34%)
Cured and Preserved Meats	108	**1900**	105 (97%)	3 (3%)	**1350**	100 (93%)
Breaded meat and poultry	26	**735**	17 (65%)	9 (35%)	**470**	10 (39%)

	n	Regional Target (mg Na/100 g)	% Meeting Regional Target	% Exceeding Regional Target	Lower Target (mg Na/100 g)	% Meeting Lower Target
Bread	95	**600**	87 (92%)	8 (8%)	**400**	36 (38%)
Mayonnaise	20	**1050**	19 (95%)	1 (5%)	**670**	12 (60%)
Cookies and Sweet Cookies	190	**485**	145 (76%)	45 (24%)	**265**	57 (30%)
Flavored Cookies and Crackers	3	**1340**	3 (100%)	0 (0%)	**700**	2 (67%)
Cakes	127	**400**	79 (62%)	48 (38%)	**205**	15 (12%)
Breakfast Cereals	112	**630**	97 (87%)	15 (13%)	**500**	78 (70%)
Butter	28	**800**	20 (71%)	8 (29%)	**500**	6 (22%)
Snacks	152	**900**	111 (73%)	41 (27%)	**530**	58 (38%)
Pasta and Noodles, uncooked	46	**1921**	45 (98%)	1 (2%)	**1333**	44 (96%)
Pasta and Noodles, as consumed	14	**640**	14 (100%)	0 (0%)	**440**	13 (93%)
Seasonings for Side and Main Dishes	51	**33100**	40 (78%)	11 (22%)	**9100**	40 (79%)
Meat and Fish Seasonings	20	**23000**	19 (95%)	1 (5%)	**21775**	19 (95%)
Bouillon Cubes and Powders	6	**20500**	3 (50%)	3 (50%)	**18000**	3 (50%)
**Barbados**	**1201**		**979 (82%)**	**220 (18%)**		**496 (41%)**
Wet and Dry Soups	80	**360**	54 (68%)	26 (33%)	**306**	44 (55%)
Noodles in Broth	0	**430**	0 (0%)	0 (0%)	**360**	0 (0%)
Meats and Sausages	139	**1210**	118 (85%)	21 (15%)	**690**	22 (16%)
Cured and Preserved Meats	10	**1900**	10 (100%)	0 (0%)	**1350**	1 (10%)
Breaded meat and poultry	17	**735**	16 (94%)	1 (6%)	**470**	4 (24%)
Bread	100	**600**	89 (89%)	11 (11%)	**400**	33 (33%)
Mayonnaise	30	**1050**	27 (90%)	3 (10%)	**670**	10 (34%)
Cookies and Sweet Cookies	108	**485**	95 (88%)	13 (12%)	**265**	37 (35%)
Flavored Cookies and Crackers	52	**1340**	51 (98%)	1 (2%)	**700**	26 (50%)
Cakes	129	**400**	62 (48%)	67 (52%)	**205**	15 (12%)
Breakfast Cereals	113	**630**	102 (90%)	11 (10%)	**500**	80 (71%)
Butter	50	**800**	47 (94%)	3 (6%)	**500**	15 (30%)
Snacks	202	**900**	150 (74%)	52 (26%)	**530**	64 (32%)
Pasta and Noodles, uncooked	49	**1921**	47 (96%)	2 (4%)	**1333**	42 (86%)
Pasta and Noodles, as consumed	8	**640**	8 (100%)	0 (0%)	**440**	8 (100%)
Seasonings for Side and Main Dishes	18	**33100**	18 (100%)	0 (0%)	**9100**	17 (95%)

	n	Regional Target (mg Na/100 g)	% Meeting Regional Target	% Exceeding Regional Target	Lower Target (mg Na/100 g)	% Meeting Lower Target
Meat and Fish Seasonings	70	**23000**	68 (97%)	2 (3%)	**21775**	68 (97%)
Bouillon Cubes and Powders	26	**20500**	17 (65%)	9 (35%)	**18000**	10 (39%)

Data presented as n (%).
